# Targeting the Golgi apparatus enhances PD-L1 blockade and synergizes with oxaliplatin to improve immunotherapy efficacy

**DOI:** 10.1016/j.jbc.2026.111343

**Published:** 2026-03-04

**Authors:** Haohuan Li, Chao Cui, Chenglu Sun, Ziyu Chen, Dengfeng Gao, Peng Yuan, Shibo Tian, Qin Zhong, Funeng Xu, Xiaoxia Liang, Long Jin, Keren Long, Lu Lu, Juan Deng, Jiaxue Cao, Xiaolan Fan, Fanli Kong, Chengdong Wang, Desheng Li, Zhiyong Qian, Mingzhou Li

**Affiliations:** 1 College of Veterinary Medicine, Sichuan Agricultural University, Chengdu, Sichuan, China; 2 College of Animal Science and Technology, Sichuan Agricultural University, Chengdu, Sichuan, China; 3 Key Laboratory of SFGA on Conservation Biology of Rare Animals, Giant Panda National Park, Chengdu, Sichuan, China; 4 Department of Biotherapy, Cancer Center and State Key Laboratory of Biotherapy, West China Hospital, Sichuan University, Chengdu, Sichuan, China; 5 College of Life Science, China West Normal University, Nanchong, Sichuan, China

**Keywords:** PD-L1 blockade, Golgi apparatus targeting, glycosylation, exosome, immunogenic cell death

## Abstract

Immune checkpoint blockade targeting programmed death ligand-1 (PD-L1) has emerged as a cornerstone of cancer immunotherapy, yielding durable responses in subsets of patients across multiple malignancies. However, clinical outcomes remain limited because of incomplete blockade, low tumor immunogenicity, and poor targeting specificity. Here, we report the development of a chondroitin sulfate–modified liposomal formulation (OPCR-Lip) designed to achieve comprehensive PD-L1 blockade while reprogramming the tumor microenvironment to enhance immune activation. OPCR-Lip binds membrane-bound PD-L1, disrupts PD-L1 glycosylation, and inhibits exosomal PD-L1 secretion by damaging the Golgi apparatus, thereby mitigating immunosuppressive signaling. Codelivery of oxaliplatin further promotes immunogenic cell death, enhancing tumor immunogenicity and sustaining antitumor immunity in 4T1 breast tumor–bearing mice. The formulation’s therapeutic precision was evaluated through circadian rhythm–based dosing, cross-species *in vitro* validation (canine and human breast cancer cells), and *in vivo* efficacy across melanoma and lung cancer models. Collectively, this study presents a promising therapeutic platform that augments PD-L1 blockade, broadens its clinical applicability, and improves treatment safety and effectiveness in solid tumors.

Cancer immunotherapy has reshaped oncologic treatment by harnessing the immune system to selectively eliminate tumor cells (1). Among immunotherapeutic strategies, immune checkpoint blockade has shown particular success, with programmed death ligand-1 (PD-L1) inhibition delivering significant clinical benefits in a range of solid tumors (2). Current PD-L1-targeted therapies primarily block PD-1–PD-L1 interactions by binding to PD-L1 at the cell surface (3).

However, PD-L1’s immunosuppressive function in tumor cells involves more than membrane expression; it relies on a complex biosynthetic and trafficking pathway closely associated with the Golgi apparatus. PD-L1 is glycosylated in the Golgi and then trafficked to the plasma membrane, where it binds PD-1 to suppress T-cell activity (4, 5). In addition, a fraction of PD-L1 is packaged into exosomes *via* endosomal pathways linked to the Golgi (6, 7), contributing to systemic immunosuppression (8, 9). Therefore, to effectively disrupt PD-L1-mediated immune evasion, it is necessary to target multiple stages of its maturation and trafficking, including glycosylation, membrane localization, and exosomal release. Building upon existing peptide inhibitor–based approaches that block surface PD-L1, we introduce an enhanced strategy that disrupts Golgi function within tumor cells to simultaneously impair PD-L1 glycosylation (10) and inhibit its exosomal secretion (11). This dual approach enables more comprehensive suppression of PD-L1 immunosuppressive activity.

To further potentiate PD-L1-targeted therapy (12), we incorporated oxaliplatin (OXA), a chemotherapeutic agent known to induce immunogenic cell death (ICD), thereby enhancing tumor immunogenicity and converting immunologically "cold" tumors into "hot" ones more responsive to checkpoint blockade (13).

In this study, we engineered a multifunctional liposomal delivery system coencapsulating ^D^PPA (a PD-L1-binding peptide) (14), retinoic acid (RA, to disrupt the Golgi apparatus), and OXA. This formulation targets multiple facets of PD-L1 function while stimulating ICD in 4T1 breast cancer cells to promote T-cell infiltration and immune activation. We further evaluated the therapeutic potential of OPCR-Lip through mechanistic studies, including single-cell transcriptomic analysis, toxicity profiling in normal tissues, circadian rhythm–based dosing optimization, and efficacy assessments across diverse tumor types (melanoma and lung cancer) and species (canine and human breast cancer cells). This comprehensive evaluation underscores the translational promise of this platform for improving PD-L1 blockade–based immunotherapy.

## Results

### PCR-Lip disrupts the structure of the Golgi apparatus and impairs its function

To develop a more effective tumor-specific PD-L1 blockade strategy, we engineered a liposomal delivery system modified with CR (chondroitin sulfate [CS] conjugated to RA *via* an ester bond), previously demonstrated to disrupt the Golgi apparatus (15). These liposomes were further functionalized with P^D^PPA-1, a bifunctional peptide comprising a PD-L1 inhibitory segment (^D^PPA-1) and an MMP-2-sensitive linker, yielding the final formulation termed PCR-Lip. As a control, we also prepared PCD-Lip, which lacks Golgi-disrupting capability (Fig. S1). Notably, the ^D^PPA-1-responsive design facilitates the selective release of ^D^PPA-1 in the tumor microenvironment (Fig. S2*A*), ensuring preferential accumulation in CD44^+^ tumor cells because of the enhanced affinity of CS for CD44 receptors (Fig. S2, *B* and *C*).

**Figure 1:**
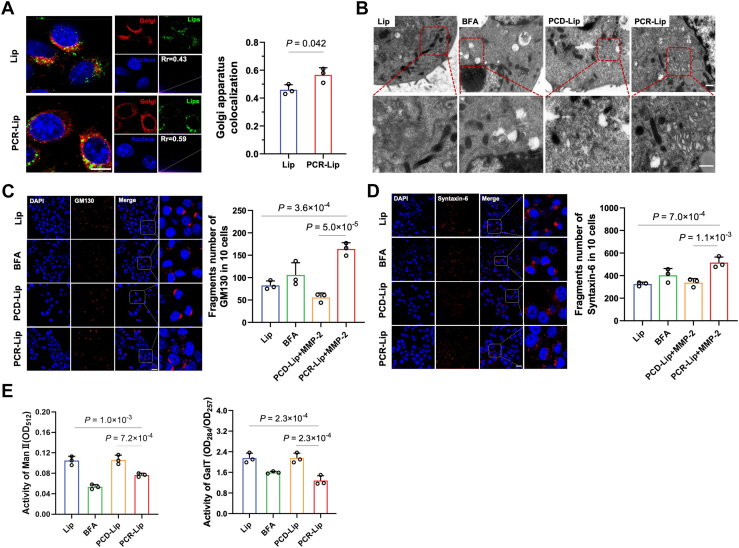
**PCR-Lip impairs the structure and function of the Golgi apparatus**. *A*, confocal microscopy images showing colocalization of FITC-labeled PCR-Lip (*green*) with the Golgi apparatus (*red*), with quantification of colocalization efficiency (*n* = 3). The scale bar represents 2 μm. *B*, TEM images of the Golgi apparatus following different treatments. PCR-Lip induced fragmentation of the Golgi ribbon into ministacks (outlined in *red*), in contrast to intact Golgi morphology in the empty liposome (Lip) group. The scale bar represents 500 nm. *C* and *D*, immunofluorescence staining and quantification of GM130 and Syntaxin-6 fragmentation in 4T1 cells (*n* = 10). PCR-Lip induced greater fragmentation than BFA, indicating substantial Golgi damage. Cells in the “PCR-Lip + MMP-2” group were treated with the PCR-Lip formulation in the presence of 2.5 μg/ml exogenous MMP-2 enzyme. The scale bar represents 25 μm. *E*, enzymatic activity of Man II and GalT in 4T1 cells following various treatments (*n* = 3). BFA, brefeldin A; GalT, galactosyltransferase; Man II, mannosidase II; TEM, transmission electron microscopy.

To investigate the Golgi-targeting capability and structural interference of PCR-Lip, we treated 4T1 breast cancer cells with a low-cytotoxicity dose of PCR-Lip (Fig. S2*D*). As expected, CS facilitated efficient Golgi targeting *via* caveolae/raft-mediated endocytosis (16) (Fig. 1*A* and Fig. S2*E*). Transmission electron microscopy (TEM) revealed that PCR-Lip treatment led to disassembly of the Golgi ribbon structure into fragmented ministacks (Fig. 1*B*). Immunofluorescence analysis further confirmed significant fragmentation of GM130 and Syntaxin-6, markers of the *cis*- and *trans*-Golgi networks, respectively (Fig. 1, *C* and *D*), indicating profound structural disruption. This disorganization was more severe than that induced by brefeldin A, a known Golgi-disrupting agent. Consistent with structural impairment, the enzymatic activities of two key Golgi-resident enzymes, mannosidase II (Man II) and UDP-galactosyltransferase (GalT), were significantly reduced following PCR-Lip treatment (Fig. 1*E*). These findings confirm that PCR-Lip effectively delivers RA to the Golgi apparatus, disrupts its structure, and compromises its functional integrity.

### PCR-Lip inhibits PD-L1 glycosylation, membrane localization, and exosomal secretion

**Figure 2:**
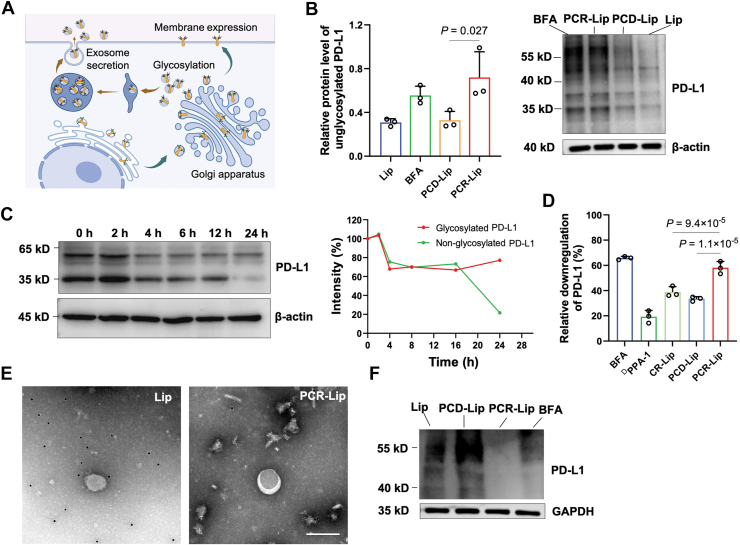
**PCR-Lip blocks PD-L1 expression in 4T1 cells**. *A*, schematic of PD-L1 synthesis, including glycosylation, membrane localization, and exosomal secretion. *B*, Western blot analysis of PD-L1 expression in 4T1 cells treated with different liposomal formulations. *C*, time-course Western blot of PD-L1 in 4T1 cells treated with PCR-Lip and actinomycin D (20 mM), which inhibits new protein synthesis. *D*, flow cytometry analysis and quantification of membrane PD-L1 in 4T1 cells across treatment groups, normalized to Lip control (*n* = 3). *E*, TEM images of PD-L1-labeled exosomes isolated from 4T1 cells, stained with 2% vanadium solution. The scale bar represents 200 nm. *F*, Western blot analysis of PD-L1 in exosomes isolated from 4T1 cells treated with different formulations. GAPDH was used as a loading control. Data are presented as means ± SD. PD-L1, programmed death ligand-1; TEM, transmission electron microscopy.

To assess whether PCR-Lip can comprehensively block PD-L1 at multiple regulatory levels, namely glycosylation, membrane presentation, and exosome-mediated secretion (Fig. 2*A*), we incubated liposomes with 4T1 cells. PCR-Lip treatment resulted in a marked reduction in PD-L1 glycosylation (Fig. 2*B*), likely because of interference with the glycan modifications of four conserved asparagine (N) residues (N35, N191, N199, and N218), potentially altered to glutamine (Q) residues (Fig. S3). Notably, the stability of nonglycosylated PD-L1 decreased over time following PCR-Lip exposure (Fig. 2*C*), suggesting that glycosylation disruption compromises PD-L1 protein stability.

Flow cytometric analysis revealed significantly lower membrane-associated PD-L1 in the PCR-Lip group compared with ^D^PPA-1 and CR-Lip controls (*p* < 0.01), indicating that ^D^PPA-1 conjugation efficiently inhibits PD-L1 surface expression (Fig. 2*D*). To evaluate effects on exosomal PD-L1 secretion, we isolated exosomes from treated 4T1 cells (Fig. S4). TEM with PD-L1 immunogold labeling confirmed substantially reduced PD-L1 content in exosomes after PCR-Lip treatment (Fig. 2, *E* and *F*). In addition, PCR-Lip markedly decreased the expression of membrane transport proteins Abcd1, Abcd3, and Tap1 (17, 18, 19), which are regulated *via* exosome-associated pathways (20) (Fig. S5). Collectively, these findings indicate that PCR-Lip simultaneously disrupts multiple steps of PD-L1 regulation, effectively blocking its immunosuppressive functions in tumor cells.

### OPCR-Lip enhances antitumor immunity *via* induction of ICD

To further augment antitumor efficacy beyond PD-L1 blockade, we engineered OPCR-Lip by coencapsulating the chemotherapeutic agent OXA within PCR-Lip to induce ICD. OPCR-Lip displayed a uniform hydrodynamic diameter of 239.0 ± 1.10 nm (Fig. S6*A*) and exhibited excellent *in vitro* stability (Fig. S6*B*), facilitating tumor accumulation through the enhanced permeability and retention effect. Its favorable hemocompatibility was confirmed by low hemolysis rates in rabbit whole blood at OXA concentrations ranging from 1 to 80 μg/ml (Fig. S6*C*). Moreover, the ^D^PPA-1 peptide was conjugated to an MMP-2-sensitive linker (P^D^PPA-1), ensuring targeted and efficient release of OXA at tumor sites (Fig. S6*D*).

**Figure 3:**
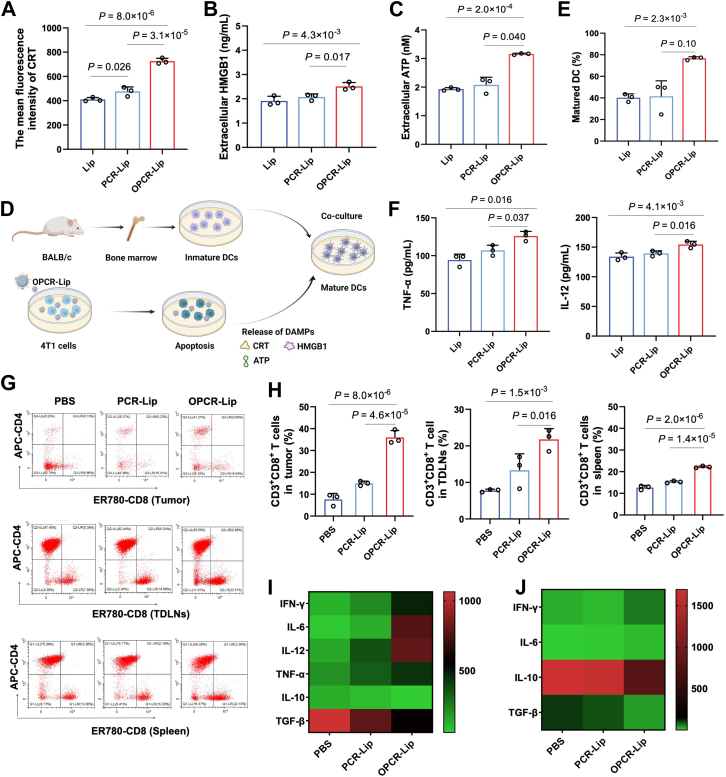
**Induction of ICD and activation of immune cells *via* OPCR-Lip**. *A*, flow cytometric quantification of CRT exposure in 4T1 cells (*n* = 3). *B*, HMGB1 release from 4T1 cells measured by ELISA (*n* = 3). *C*, ATP secretion in 4T1 cell supernatants quantified using an ATP detection kit (*n* = 3). *D*, schematic of *in vitro* maturation of bone marrow–derived DCs. *E*, proportion of mature DCs following coculture with pretreated 4T1 cells, assessed by flow cytometry (*n* = 3). *F*, ELISA quantification of TNF-α and IL-12 in DC culture supernatants (*n* = 3). *G* and *H*, flow cytometry images and quantification of CD8^+^ T cells in tumor, spleen, and TDLNs (*n* = 3). *I*, ELISA quantification of cytokines in tumor tissues. *J*, serum cytokine levels in treated mice (*n* = 3). Data are presented as means ± SD. CRT, calreticulin; DC, dendritic cell; HMGB1, high mobility group box 1; ICD, immunogenic cell death; OPCR, a free-drug solution of OXA and PCR; TNF-α, tumor necrosis factor alpha.

By incorporating OXA into PCR-Lip, we aimed to trigger ICD and enhance immune activation. Compared with PCR-Lip and PBS control, OPCR-Lip significantly promoted ICD in 4T1 cells (Fig. 3, *A–C*). Notably, OPCR-Lip synergistically combined OXA-induced ICD with PCR-Lip–mediated endoplasmic reticulum stress to elevate calreticulin (CRT) exposure (*p* = 0.01; Fig. 3*A*). This synergism is crucial, as cancer cells undergoing ICD facilitate dendritic cell (DC) maturation and antigen presentation, thereby activating CD8^+^ cytotoxic T lymphocytes (CTLs). Indeed, OPCR-Lip markedly promoted DC maturation *in vitro* upon coculture with pretreated 4T1 cells (Fig. 3, *D* and *E*) and *in vivo* in 4T1 tumor–bearing mice (Fig. S7). In addition, OPCR-Lip–matured DCs secreted higher levels of tumor necrosis factor-α and interleukin (IL)-12, cytokines essential for CTL activation (Fig. 3*F*) (21, 22).

To assess the immunomodulatory effects of OPCR-Lip *in vivo*, we compared its performance with PCR-Lip and PBS. OPCR-Lip significantly increased CD8^+^ CTL infiltration into tumors, spleens, and tumor-draining lymph nodes (TDLNs) (Fig. 3, *G* and *H*). Tumors from OPCR-Lip–treated mice also showed elevated levels of proinflammatory cytokines (IL-6, IL-12, and interferon gamma [IFN-γ]) and decreased immunosuppressive cytokines (transforming growth factor-β and IL-10), reflecting a shift toward a highly active antitumor immune microenvironment (Fig. 3*I*). Similar cytokine trends were observed systemically in mouse serum (Fig. 3*J*). Collectively, these results demonstrate that OPCR-Lip effectively induces ICD and reprograms the immune microenvironment, promoting CD8^+^ CTL infiltration and cytokine-mediated antitumor responses.

### *In vivo* antitumor efficacy of OPCR-Lip

**Figure 4:**
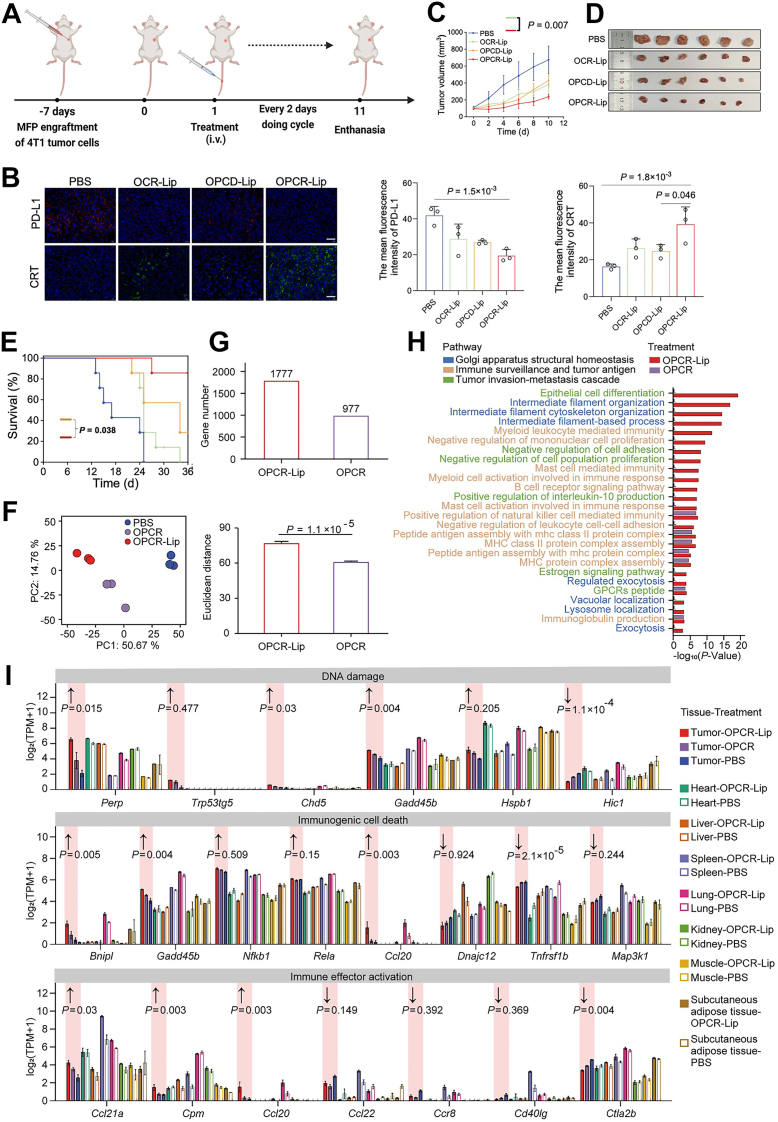
***In vivo* antitumor efficacy of OPCR-Lip**. *A*, schematic illustration of the treatment regimen administered to 4T1 tumor–bearing mice. *B*, representative immunofluorescence images showing PD-L1 and CRT expression in tumor sections post-treatment. The scale bar represents 40 μm. *C*, tumor growth curves of 4T1-bearing mice treated with various formulations *via* intravenous injection (*n* = 6). *D*, representative images of tumors excised from each treatment group. *Solid black lines* separate the groups, and a ruler (millimeter) indicates scale. *E*, Kaplan–Meier survival curves of 4T1 tumor–bearing mice following different treatments (*n* = 6). *F*, principal component analysis (PCA) of tumor gene expression profiles *(left*). Corresponding bar graphs (*right*) depict the Euclidean distances from the control group to the OPCR-Lip and OPCR-treated groups. *G*, quantification of differentially expressed genes (DEGs) following OPCR-Lip or OPCR treatment. *H*, functional enrichment analysis of DEGs identified in tumor tissues after treatment with OPCR-Lip or OPCR. *I*, expression patterns of key signature genes across tumor and nontumor tissues. *Arrows* denote the direction of gene expression changes and associated functional enhancements elicited by OPCR-Lip or OPCR. All data are presented as mean ± SD. CRT, calreticulin; OPCR, a free-drug solution of OXA and PCR; PD-L1, programmed death ligand-1.

To comprehensively evaluate the therapeutic potential of OPCR-Lip, we conducted *in vivo* studies using 4T1 tumor–bearing BALB/c mice (Fig. 4*A*). One week postinoculation of 4T1 cells into the mammary fat pad, mice were intravenously administered OPCR-Lip (containing 5 mg/kg OXA) every 2 days for a total of five doses. OPCR-Lip was compared against five control groups: PBS, O-Lip (OXA-loaded liposomes), OCR-Lip (OPCR-Lip lacking P^D^PPA-1), PCR-Lip, and OPCD-Lip (OXA-loaded, Golgi-inactive liposomes).

Among all groups, OPCR-Lip demonstrated the most potent therapeutic effect, evidenced by robust ICD induction and minimal PD-L1 expression within the tumor microenvironment (Figs. 4*B* and S8). Unlike prior reports (23), no PD-L1 upregulation was observed with O-Lip, likely because of differences in dosing (Fig. S8*A*). Notably, OPCR-Lip significantly enhanced CRT exposure compared with OPCD-Lip (*p* < 0.05), supporting the synergistic role of OXA-induced ICD and Golgi-targeted PD-L1 blockade (Fig. 4*B*), consistent with Fig. 3*A*. Histological analyses revealed extensive tumor necrosis, reduced proliferation (*via* TUNEL assay), and elevated apoptosis (*via* Ki67 staining) in the OPCR-Lip group (Fig. S9*A*). These cellular changes translated into markedly reduced tumor growth (Fig. 4, *C* and *D* and Fig. S9*B*) and prolonged survival (Fig. 4*E* and Table S1).

Importantly, no damage was observed in vital organs during the treatment period, indicating that OPCR-Lip exhibited excellent safety throughout the short-term treatment course. Mice treated with OPCR-Lip showed no significant changes in body weight (*p* > 0.05) (Fig. S9*C*) and exhibited no histological damage in vital organs, including heart, liver, lung, kidney, and spleen (Fig. S9*D*), confirming favorable biosafety profiles.

To further delineate the transcriptional mechanisms underlying the potent antitumor effects of OPCR-Lip, we performed bulk RNA-Seq on tumor tissues harvested from mice treated with OPCR-Lip, OPCR (a free-drug solution of OXA and PCR), or PBS as a control. Notably, OPCR-Lip treatment resulted in the smallest tumor volumes (Fig. S10). Following this observation, tumors treated with OPCR-Lip exhibited significantly greater transcriptional divergence from PBS controls than those treated with OPCR, as evidenced by increased Euclidean distances in the two-dimensional principal component analysis and a higher number of differentially expressed genes (DEGs) (Fig. 4, *F* and *G*). These results indicate that OPCR-Lip elicits a more pronounced transcriptional response in the tumor microenvironment.

Functional enrichment analysis of OPCR-Lip–induced DEGs revealed significant upregulation of pathways related to Golgi structural homeostasis (*e*.*g*., “intermediate filament organization”) (24), immune surveillance and antigen presentation (*e*.*g*., “myeloid leukocyte-mediated immunity”) (25), and the epithelial–mesenchymal transition and metastatic progression (*e*.*g*., “epithelial cell differentiation”) (26) (Fig. 4*H*). These findings confirm that OPCR-Lip exerts broader and more potent effects on Golgi function, immune activation, and metastasis-associated processes compared with OPCR alone.

To validate the functional relevance of these transcriptional changes, we further examined the expression profiles of 21 signature genes associated with the proposed mechanisms of OPCR-Lip (Table S2). As anticipated, all observed gene expression changes aligned with the expected direction of OPCR-Lip action, reinforcing its capacity to enhance tumor immunogenicity and stimulate robust immune responses (Fig. 4*I*).

Despite the strong antitumor efficacy of OPCR-Lip, marked by stable body weight and absence of pathological damage in off-target tissues, *ex vivo* fluorescence imaging revealed a modest accumulation of the formulation outside the tumor (Fig. S11). Given the inherent risks of chemotherapy-induced toxicity, we investigated whether OPCR-Lip triggered off-target effects at the transcriptomic level. Bulk RNA-Seq was conducted on six representative organs, along with skeletal muscle and adipose tissue, both of which are essential for metabolic homeostasis and immune modulation during cancer treatment (27, 28). Consistent with histological assessments showing minimal tissue injury (Fig. S11), transcriptomic analyses revealed only minor, nonsignificant expression changes in nontarget tissues (Fig. 4*I*), highlighting the high tumor selectivity and favorable safety profile of OPCR-Lip.

### OPCR-Lip suppresses tumor growth and sustains an antitumor immune response

**Figure 5:**
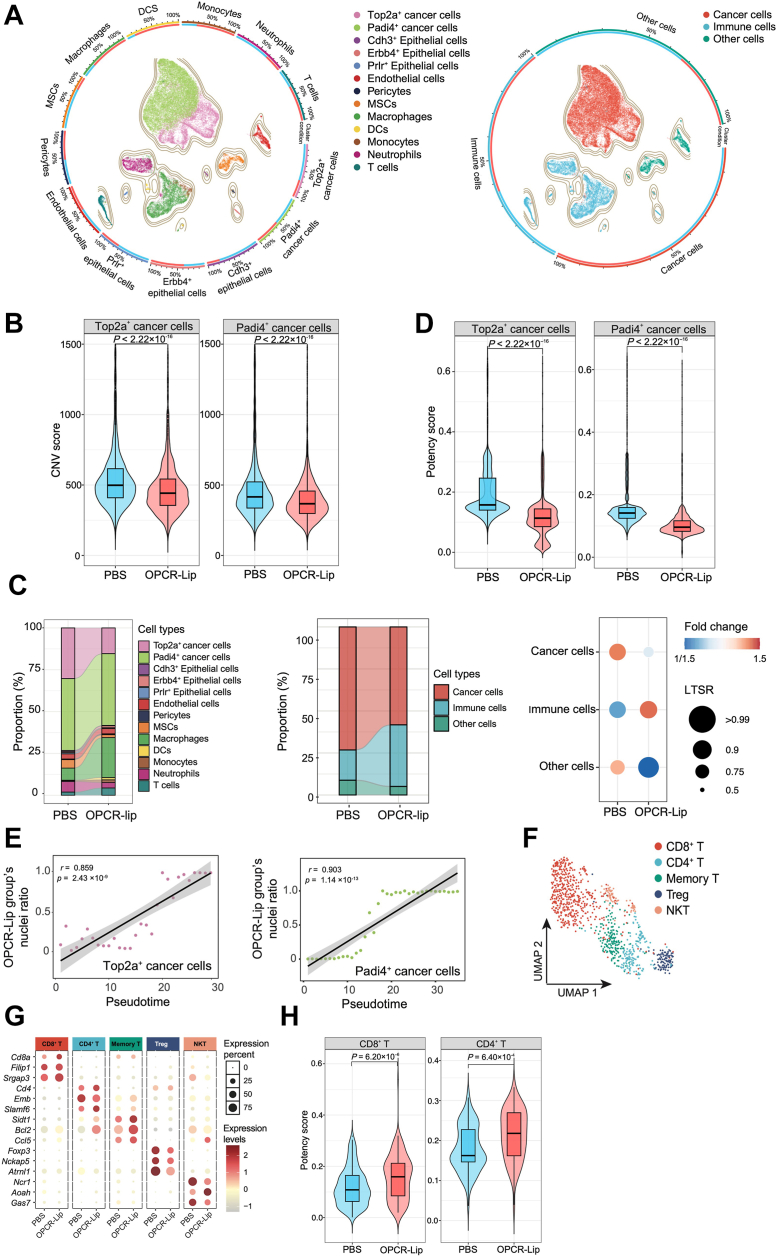
**Cell dynamics of tumor tissues in a breast cancer mouse model treated with OPCR-Lip**. *A*, UMAP plot of 32,087 nuclei. *Left*, 13 transcriptionally distinct clusters identified by marker gene expression. *Right*, annotated major cell types. *B*, violin plots showing CNV scores in malignant cells post-treatment (Wilcoxon's rank-sum test). *C*, *left*, Sankey diagram illustrating proportional changes across all 13 cell clusters following OPCR-Lip treatment. *Middle*, simplified Sankey diagram highlighting major cell type shifts. *Right*, bar plot showing relative fold changes of major cell populations post-treatment. Fold change and local true sign rate (LTSR) were calculated using a generalized linear mixed model with a Poisson outcome. *D*, violin plots of differentiation potential scores for malignant cells (Wilcoxon's rank-sum test). *E*, pseudotime analysis showing the temporal trajectory of malignant cell differentiation in OPCR-Lip–treated tumors. Pearson's correlation coefficient (*r*) and *p* value are derived from a linear model; *gray shading* indicates the 95% confidence interval. *F*, UMAP visualization of T-cell subsets. *G*, dot plot of marker gene expression profiles across identified T-cell clusters. *H*, violin plots of differentiation potential scores for CD8^+^ and CD4^+^ T cells in response to OPCR-Lip (Wilcoxon's rank-sum test). CNV, copy number variation; OPCR, a free-drug solution of OXA and PCR; UMAP, uniform manifold approximation and projection.

In cancer immunotherapy, immune cells act as effectors, whereas tumor cells not only serve as immune targets but also actively modulate immune function through diverse mechanisms. To dissect the cellular heterogeneity underlying the therapeutic effects of OPCR-Lip, we performed single-nucleus RNA-Seq (snRNA-Seq) on tumor tissues following treatment. Uniform manifold approximation and projection (UMAP) analysis of 32,087 individual nuclei identified 13 transcriptionally distinct cell clusters (Fig. 5*A*, Fig. S12, *A*, *C*, and *D*, and Table S3). Among these, two clusters—Top2a^+^ and Padi4^+^ malignant cells—exhibited marked copy number variations (CNVs), consistent with their oncogenic identity, as revealed by inferCNV analysis (Fig. S12*B*). Notably, tumors treated with OPCR-Lip showed a significant reduction in both the abundance and CNV burden of these malignant populations compared with PBS controls (Fig. S12*B*), suggesting a suppression of chromosomal instability (CIN), a major driver of tumor progression (29).

Simultaneously, OPCR-Lip treatment led to a pronounced expansion of four key immune cell populations—macrophages, DCs, monocytes, and T cells—indicating enhanced recruitment and activation of immune effectors, likely facilitated by immunogenic tumor cell death induced by chemotherapy (30) (Figs. 5*C* and S12*E*). This reshaping of the tumor microenvironment supports the observed antitumor efficacy and suggests an immune-permissive milieu conducive to sustained therapeutic response.

Tumor progression is tightly linked to the differentiation and maturation state of malignant cells (31, 32). To evaluate how OPCR-Lip modulates cancer cell plasticity, we applied CytoTrace analysis (33), which revealed significantly lower developmental potential scores for Top2a^+^ and Padi4^+^ malignant cells in OPCR-Lip–treated tumors compared with PBS-treated controls (Fig. 5*D*). Consistent with this, pseudotime trajectory analysis demonstrated a more advanced differentiation state in OPCR-Lip–treated malignant cells (Fig. 5*E*). These results suggest that OPCR-Lip not only restricts malignant cell proliferation but also pushes them toward terminal differentiation, thereby limiting their adaptability under therapeutic pressure.

Given the central role of CD8^+^ and CD4^+^ T cells in orchestrating antitumor immunity (34, 35), we next characterized these immune subsets. CytoTrace analysis showed significantly increased differentiation potential in both CD8^+^ and CD4^+^ T cells from OPCR-Lip–treated tumors compared with PBS-treated controls (Fig. 5, *F–H* and Fig. S12*F*), indicative of restored immune competence. This suggests that OPCR-Lip reverses T-cell exhaustion and reprograms them into a more functional state, thereby enhancing immune surveillance and therapeutic durability (36, 37).

Together, these findings demonstrate that OPCR-Lip not only effectively suppresses tumor proliferation but also reconfigures the tumor immune landscape, enabling durable immune responses through enhanced T-cell functionality and reduced malignant cell plasticity.

### Circadian rhythm–based optimization of OPCR-Lip administration

**Figure 6:**
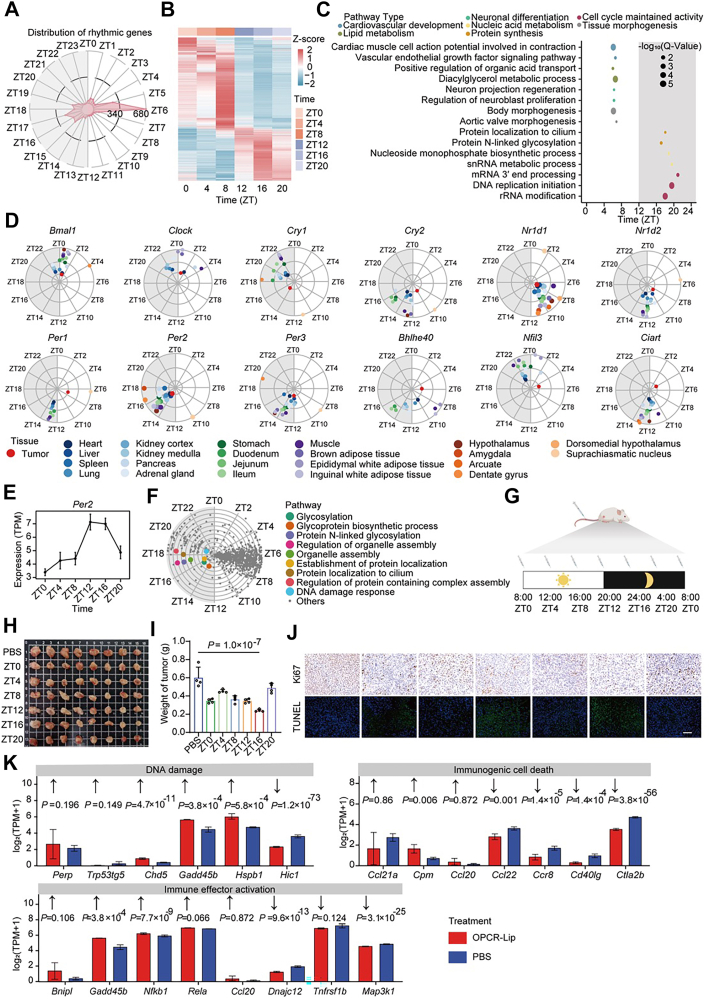
**Antitumor therapeutic effects of OPCR-Lip–based circadian clocks**. *A*, radial plot showing the peak phase distribution of cycling genes in tumor tissue. *Gray background* denotes the dark phase (ZT12–ZT24/ZT0). *B*, heatmap of cycling gene expression across 24 h. *C*, enriched functional pathways centered around ZT6 and ZT16. Dot size indicates statistical significance; color reflects pathway category. *D*, oscillation patterns of core clock genes in tumor tissues, as derived from MetaCycle. Colored dots indicate peak phases in normal tissues from healthy mice. *E*, temporal expression profile of *Per2* in tumor tissue (log_2_[1 + TPM]). *F*, peak phase distribution of enriched pathways in tumor tissue. OPCR-Lip–relevant pathways are color-highlighted. *Gray background* indicates a dark phase. *G*, *in vivo* administration schedule (*n* = 8). *H*, representative images of excised tumors. *I*, tumor weights from treated mice. *J*, TUNEL and Ki67 staining of tumor sections. The scale bar represents 50 μm. *K*, expression of marker genes in tumor tissues following ZT16 treatment. *p* Values (from edgeR) indicate significance *versus* PBS. *Arrows* represent expected expression changes as reported in prior literature. All data are presented as means ± SD. OPCR, a free-drug solution of OXA and PCR; TPM, transcripts per million.

Mounting evidence suggests that aligning chemotherapy with the body's endogenous circadian rhythms can significantly enhance therapeutic efficacy (38, 39, 40). To explore the circadian characteristics of tumors in 4T1-bearing BALB/c mice and identify an optimal treatment window for OPCR-Lip, we performed a comprehensive temporal transcriptomic analysis over a 24-h cycle. Using bulk RNA-Seq, we generated 34 datasets with four to seven biological replicates collected at 4-h intervals (Table S4). MetaCycle analysis (41) identified 3674 cycling genes within the tumor tissue. These genes exhibited a bimodal phase distribution, with peak expression clustering around ZT5–ZT6 (early afternoon) and ZT16–ZT18 (late night) (Fig. 6, *A* and *B*).

Functionally, genes peaking at ZT6 were enriched in pathways related to cardiovascular development, lipid metabolism, neuronal differentiation, and tissue morphogenesis—biological processes typically active at night in healthy mice (Fig. 6*C*). Compared with nontumor tissues from healthy mice (42), tumors exhibited significant phase shifts in the expression of core circadian regulators, particularly *Cry1* and *Cry2*. These disruptions were especially prominent in metabolically active organs, such as the liver, skeletal muscle, and adipose tissue, as well as in neural tissues, including the hypothalamus, suprachiasmatic nucleus, and dentate gyrus (Fig. 6*D*). These findings highlight circadian desynchrony in tumor tissues, likely linked to behavioral and physiological dysregulation during tumor progression (43, 44).

Building on this circadian landscape, we designed a mechanism-driven chronochemotherapy strategy for OPCR-Lip administration. DNA damage repair—a key determinant of platinum-based chemotherapy efficacy—is tightly regulated by circadian genes (45, 46, 47). Notably, *Per2* enhances both the cytotoxicity and sensitivity to OXA (48, 49), and its rhythmic peak has been shown to optimize therapeutic response in human oral squamous cell carcinoma (50). In our study, *Per2* expression peaked around ZT16 in 4T1 tumors (Fig. 6*E*). Furthermore, OPCR-Lip–targeted pathways—including glycosylation regulation, Golgi-mediated protein transport, and the DNA damage response—also peaked between ZT14 and ZT20 (Fig. 6*F*). This temporal convergence strongly supports night-time (ZT16) administration of OPCR-Lip to align with peak therapeutic target activity and *Per2*-mediated sensitivity.

To empirically determine the optimal administration window, we delivered OPCR-Lip to 4T1-bearing mice every 4 h over a 24-h period (Fig. 6*G*). Consistent with previous chronochemotherapy studies (51), OPCR-Lip exhibited time-dependent efficacy. The ZT16 treatment group demonstrated the most pronounced therapeutic benefit, including significant tumor weight reduction, stable body weight maintenance (Fig. 6, *H* and *I*, and Fig. S13), and increased tumor cell necrosis and apoptosis (Fig. 6*J*). These effects were underpinned by robust immune activation, as evidenced by transcriptomic shifts in the ZT16-treated tumors.

Markers of DNA damage (*Chd5*, *Gadd45b*, and *Hspb1*), ICD (*Nfkb1*, *Dnajc12*, and *Map3k1*), and immune effector activation (*Cpm*, *Ccr8*, and *Cd40lg*) were significantly upregulated following ZT16 administration (Fig. 6*K*), highlighting the synergistic enhancement of OXA and PD-L1 blockade during this circadian phase. While transcriptomic data elucidate the pharmacodynamic mechanisms driving time-dependent efficacy, circadian fluctuations in pharmacokinetics and drug metabolism may also contribute to the observed outcomes (52).

Collectively, these findings establish ZT16 (night-time) as the optimal administration window for OPCR-Lip, providing a mechanistic and empirical basis for circadian-timed immunochemotherapy aimed at maximizing therapeutic efficacy.

### Evaluation of the antitumor efficacy of OPCR-Lip in skin and lung cancer models

**Figure 7:**
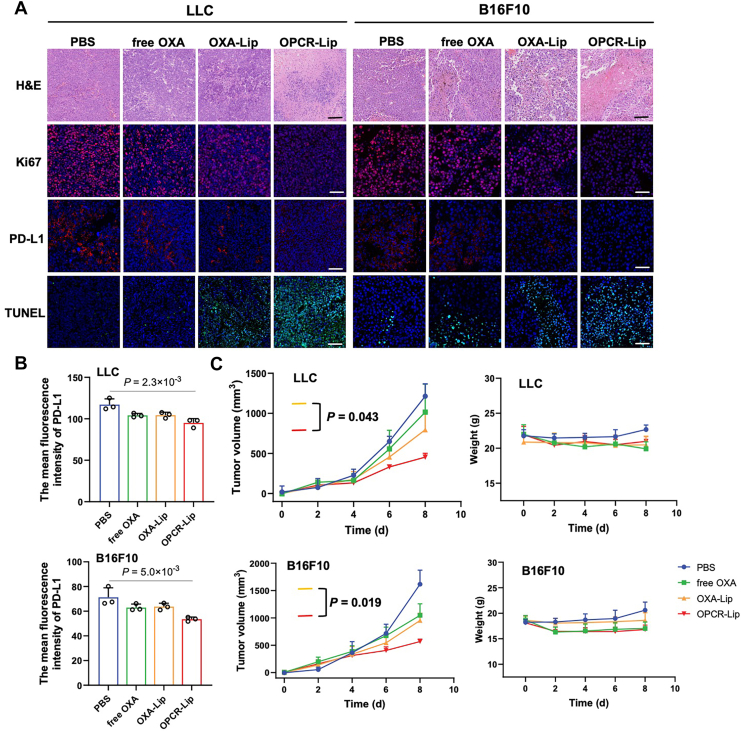
**Antitumor effects of OPCR-Lip in LLC and B16F10 tumor–bearing mice**. *A*, representative H&E, TUNEL, PD-L1, and Ki67 staining of tumor tissue sections from treated mice. The scale bar represents 50 μm. *B*, quantification of PD-L1 expression *via* immunofluorescence (n = 3). *C*, tumor growth curves and body weight changes of LLC and B16F10 tumor–bearing mice following intravenous administration of different formulations (n = 6). Data are presented as mean ± SD. LLC, Lewis lung carcinoma; OPCR, a free-drug solution of OXA and PCR; PD-L1, programmed death ligand-1.

To evaluate the broad-spectrum antitumor efficacy of OPCR-Lip against malignancies with variable PD-L1 expression (53), we investigated its effects in melanoma (B16F10) and lung carcinoma (Lewis lung carcinoma [LLC]) models. OPCR-Lip treatment markedly increased tumor necrosis, suppressed cellular proliferation, and enhanced apoptosis in both tumor types, collectively resulting in the most pronounced inhibition of tumor growth among all groups. Importantly, OPCR-Lip exhibited superior antitumor activity compared with OXA alone and OXA-Lip, indicating that its efficacy arises not only from OXA but also from the synergistic effects of other components. (Fig. 7, *A–C*).

Transcriptomic profiling of tumor tissues treated with OPCR-Lip *versus* PBS revealed distinct gene expression changes in both B16F10 and LLC models. DEGs were predominantly enriched in pathways related to immune cell proliferation (Fig. S14*A*). Furthermore, the gene expression signatures induced by OPCR-Lip were highly consistent with remodeling of the tumor immune microenvironment (Fig. S14*B*), indicating that OPCR-Lip can potentially reprogram immune cell function and restore antitumor immunity.

In addition to its immunomodulatory effects, OPCR-Lip also altered metastasis-related gene expression profiles in these highly metastatic tumors. Specifically, in B16F10 tumors, five genes were significantly modulated, including those linked to primary tumor progression (*Cdkn1a* (54), *Epha3* (55), and *Acsl3* (56)) and invasion (*Cdh1* (57)) (Fig. S14*B*). In LLC tumors, three genes associated with epithelial–mesenchymal transition—a key driver of tumor cell invasiveness—were identified: *Tgfbi* (58), *Mmp9* (59), and *Mmp10* (60) (Fig. S14*B*). These findings indicate that OPCR-Lip suppresses metastasis by targeting multiple steps of the metastatic cascade in both tumor models.

Together, these results demonstrate that OPCR-Lip exerts potent antitumor activity in B16F10 and LLC tumors through a dual mechanism: activating immune responses and inhibiting metastatic progression.

### OPCR-Lip demonstrates efficacy in both canine and human breast cancer cells

While OPCR-Lip has shown therapeutic efficacy in murine models, a considerable translational gap remains before it can be advanced to clinical application. Bridging this gap requires rigorous preclinical validation in larger animal models, such as canines, followed by well-controlled human clinical trials. To this end, we investigated the ability of OPCR-Lip to disrupt the Golgi apparatus and induce ICD in breast cancer cell lines derived from dogs (CMT-7364) and humans (MCF-7).

**Figure 8:**
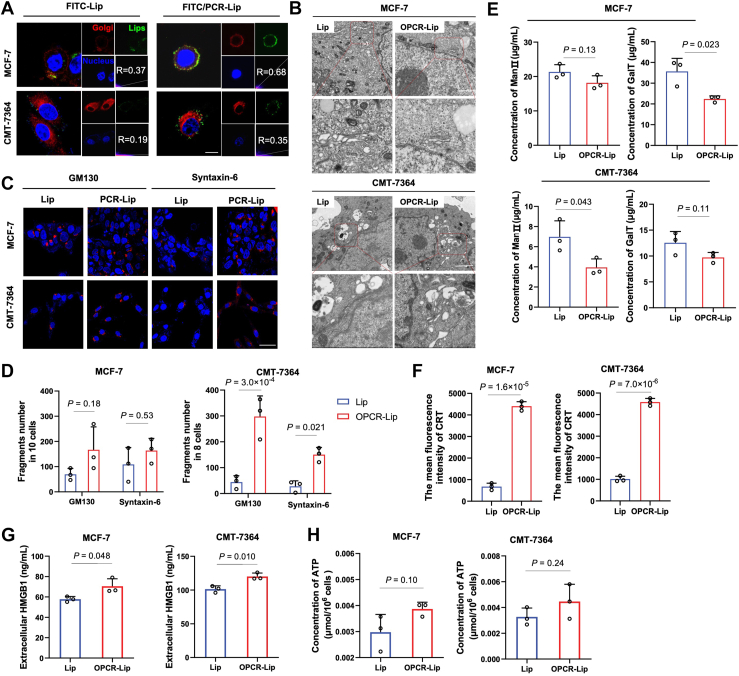
**Disruption of the Golgi apparatus and induction of ICD by OPCR-Lip in canine and human breast cancer cells**. *A*, confocal images showing colocalization of FITC/PCR-Lip (*green*) with the Golgi apparatus (*red*) in MCF-7 (human) and CMT-7364 (canine) cells, with quantification of colocalization efficiency (*n* = 3). The scale bar represents 8 μm. *B*, TEM images of the Golgi apparatus following treatment with various formulations. The scale bar represents 500 nm. *C* and *D*, immunofluorescence staining and quantification of GM130 and Syntaxin-6 fragments in MCF-7 (*n* = 10) and CMT-7364 (*n* = 8) cells. The scale bar represents 25 μm. *E*, concentrations of Man II and GalT following treatment with different formulations (*n* = 3). *F*, flow cytometry quantification of mean fluorescence intensity of CRT (*n* = 3). *G*, ELISA-based quantification of HMGB1 release (*n* = 3). *H*, ATP secretion levels in culture supernatants measured using an ATP detection kit (n = 3). All results are presented as means ± SD. CRT, calreticulin; GalT, galactosyltransferase; HMGB1, high mobility group box 1; ICD, immunogenic cell death; Man II, mannosidase II; OPCR, a free-drug solution of OXA and PCR; TEM, transmission electron microscopy.

Confocal microscopy revealed that OPCR-Lip accumulated within the Golgi apparatus in both CMT-7364 and MCF-7 cells (Fig. 8*A*), and TEM confirmed that it disrupted both *cis*- and *trans*-Golgi compartments (Fig. 8, *B–D*). Correspondingly, OPCR-Lip treatment significantly suppressed the activity of Man II in CMT-7364 cells and GalT in MCF-7 cells (Fig. 8*E*), indicating conserved Golgi-targeting specificity across species.

In addition to Golgi disruption, OPCR-Lip triggered the release of key damage-associated molecular patterns, including CRT from both CMT-7364 and MCF-7 cells, and high mobility group box 1 (HMGB1) from CMT-7364 cells. However, no significant ATP release was observed in either cell line, which may be attributed to dose-dependent effects of the ICD inducer (Fig. 8, *F–H*).

Collectively, these findings demonstrate that OPCR-Lip retains cross-species efficacy in targeting the Golgi apparatus and inducing hallmark ICD events in both canine and human breast cancer cells, supporting its potential as a translationally viable therapeutic candidate for future clinical evaluation.

## Discussion

Immune checkpoint inhibitors targeting PD-L1 have revolutionized cancer therapy, demonstrating potent and durable antitumor responses across a wide range of malignancies. Several monoclonal antibodies, such as atezolizumab, durvalumab, and avelumab, have received approval from the US Food and Drug Administration for treating various cancers, with additional indications likely to follow soon (3). Despite these advances, therapeutic resistance remains a significant obstacle in certain patient populations and tumor types, often driven by systemic immunotoxicity, incomplete PD-L1 blockade, or insufficient T-cell responses because of poor tumor immunogenicity (61). When interpreting these promising results, it is important to note that this work did not directly compare the nanoparticle platform with free OXA, a standalone PD-L1 inhibitor, or their unencapsulated combination—an aspect that warrants future investigation to clarify its therapeutic profile. Moreover, the PD-L1 inhibitor used here is a peptide, distinct from clinically approved monoclonal antibodies; thus, a comprehensive efficacy comparison would require parallel assessment of key parameters such as binding affinity and blockade efficiency.

In this study, we developed a liposomal platform (PCR-Lip) codelivering the Golgi-disrupting agent CR and the anti-PD-L1 peptide ^D^PPA, designed to achieve comprehensive and tumor-specific PD-L1 blockade. Given that the synthesis achieved coupling efficiencies of 1.36% and 2.14% (attributable to steric hindrance and the effects of the reaction medium), optimizing these yields will be a critical consideration for scalable production. Although the current yields did not compromise batch consistency, strategies such as phase-transfer catalysis (62) or enzymatic pretreatment (63) offer promising routes for improvement. To enhance tumor targeting, we functionalized the liposomes with CS in an MMP-2-sensitive manner (64), enabling selective accumulation in CD44^+^ tumor cells. This PD-L1-independent targeting strategy ensures sustained delivery efficacy upon repeated administration. Importantly, the dual-phase release mechanism of the liposomes enables sequential drug delivery in both extracellular and intracellular compartments of the tumor microenvironment, allowing for the inhibition of membrane-bound PD-L1 on the cell surface and intracellular PD-L1 glycosylation and exosomal secretion *via* Golgi apparatus disruption. To further potentiate antitumor immunity, we integrated OXA, a known inducer of ICD, into the formulation to generate OPCR-Lip. This enhanced construct promoted the maturation of DCs both *in vitro* and *in vivo*. In the 4T1 murine tumor model, OPCR-Lip not only inhibited tumor progression but also reprogrammed T cells to sustain robust and durable immune responses. As previously reported, CIN promotes tumor progression through CNVs and genomic instability (29). The ability of OPCR-Lip to reduce CNVs and stabilize the genome may directly counteract CIN-driven tumor evolution. We also conducted preliminary investigations into circadian rhythm–based administration schedules in the 4T1 model to optimize therapeutic outcomes. Moreover, OPCR-Lip demonstrated potent antitumor efficacy in additional models, including B16F10 and LLC tumor–bearing mice. It also exhibited Golgi-targeting capacity and ICD-inducing effects in both human and canine breast tumor cells, highlighting its translational potential across diverse cancer types and species. In summary, our work presents OPCR-Lip as a multifaceted and improved immune checkpoint blockade strategy. It achieves dual-phase PD-L1 inhibition, targeting both extracellular and intracellular PD-L1, and simultaneously enhances tumor immunogenicity, offering broad therapeutic promise for cancer immunotherapy.

## Experimental procedures

### Mice

Female BALB/c (6 weeks old) and male C57BL/6J (6 weeks old) mice were purchased from Dashuo Biotechnology. All mice were housed under standard laboratory conditions, including a 12-h light–dark cycle, an ambient temperature of 25 ± 2 °C, and a humidity level of 60 ± 10%. Mice were euthanized when tumor volumes reached 2000 mm^3^. All animal procedures complied with the Regulations for the Administration of Affairs Concerning Experimental Animals and were approved by the Animal Ethics and Welfare Committee of Sichuan Agricultural University (permit no.: 20250678).

### Cell lines and cell culture

The 4T1 (murine breast cancer), B16F10 (murine melanoma), LLC (murine lung carcinoma), and MCF-7 (human breast cancer) cell lines were obtained from the Shanghai Institutes for Biological Sciences, Chinese Academy of Sciences. Canine mammary carcinoma cells (CMT-7364) were provided by the College of Veterinary Medicine, China Agricultural University. All cells were cultured at 37 °C in a humidified atmosphere with 5% CO_2_. 4T1 and B16F10 cells were maintained in RPMI1640 medium; LLC and CMT-7364 in Dulbecco's modified Eagle's medium; both media were supplemented with 10% fetal bovine serum and 1% penicillin–streptomycin. DCs were isolated from BALB/c mice and cultured in RPMI1640 with 10% fetal bovine serum and antibiotics.

### Antibodies and reagents

For flow cytometry assays, CD11c-PE and CD86-FITC antibodies were procured from eBioscience, whereas the anti-CRT antibody (CRT Rabbit Monoclonal) was obtained from StressMarq. Antibodies for CD80-APC, CD3e-APC, CD4-PE, CD8a-BB515, GM130, and Syntaxin-6 were purchased from BD Pharmingen. Additional antibodies, including vascular endothelial growth factor, transforming growth factor-β, PD-L1, IL-10, IFN-γ, Alexa Fluor 594-conjugated, and Alexa Fluor 488-conjugated secondary antibodies, were obtained from Abcam. ATP assay kits were sourced from Beyotime, and ELISA kits were sourced from Elabscience. CS (molecular weight = 6000) and RA were obtained from Sigma–Aldrich. OXA was purchased from Meilune Biological, and the MMP-2-sensitive peptide P^D^PPA-1 (sequence: CPLGVRG-NYSKPTDRQYHF) was custom-synthesized by Guotai Biotechnology. A Golgi extraction kit was sourced from BestBio. All other chemicals were of analytical reagent grade.

### Synthesis and characterization of OPCR-Lip

CR was synthesized according to a previously reported protocol (15). Briefly, CR (0.02 mmol) was dissolved in PBS, followed by the dropwise addition of 1-ethyl-3-(3-dimethylaminopropyl)carbodiimide and sulfo-*N*-hydroxysulfosuccinimide, and stirred at room temperature for 40 min. A PBS solution of N-(2-aminoethyl) maleimide hydrochloride (0.04 mmol) was then added, and the mixture was stirred for 24 h. The reaction product was dialyzed and lyophilized to obtain carboxyl-reactive maleimide (CR-MAL).

To prepare CD-MAL, CS (0.2 mmol) was dissolved in formamide and slowly added to a solution of deoxycorticosterone acetate (481 mg, 1.6 mmol) in formamide. After stirring at room temperature for 48 h, the CD conjugate was isolated and further reacted with MAL to yield CD-MAL using the same procedure as for CR-MAL. The structures of CR-MAL and CD-MAL were confirmed *via* ^1^H NMR spectroscopy.

OCR-Lip nanoparticles were fabricated *via* the film dispersion/postinsertion method. Briefly, Lipoid S100 and cholesterol were dissolved in chloroform, and the organic solvent was evaporated at 55 °C to form a lipid film. The film was hydrated with a 5% glucose solution containing OXA and CR-MAL, followed by sonication for 10 min to obtain OCR-Lip. The liposome solution was then ultrafiltered to remove free OXA. P^D^PPA-1 was dissolved in 0.01 M phosphate buffer (0.2 ml) and added dropwise to the OCR-Lip dispersion at a molar ratio of peptide to maleimide groups of 18:10. The mixture was stirred at 4 °C for 12 h. The reaction product was dialyzed to remove unreacted P^D^PPA-1 for 12 h to obtain OPCR-Lip. The coupling efficiencies of P^D^PPA-1 were obtained by HPLC (14). OPCD-Lip was prepared using a similar procedure.

Nanoparticle size, polydispersity index, and zeta potential were characterized by dynamic light scattering (Zetasizer Nano ZS90) and TEM (JEM-2100Plus). Encapsulation efficiency (%), drug loading (%) of OXA, and ^D^PPA-1 conjugation efficiency were quantified by HPLC (Agilent).

### Golgi colocalization and disruption assay

Colocalization of liposomes with the Golgi apparatus was assessed by confocal laser scanning microscopy (CLSM). Cells were seeded in confocal dishes and treated with formulations for 2 h. The Golgi apparatus was stained using BODIPY TR (10 mM Hepes), and nuclei were stained with 4′,6-diamidino-2-phenylindole. Images were captured using CLSM (Nikon A1-90i) and analyzed with ImageJ (National Institutes of Health, Bethesda, MD) using the Colocalization Threshold plugin.

To evaluate Golgi disruption, cells were treated with various formulations for 24 h and immunostained with GM130 and Syntaxin-6 primary antibodies, followed by fluorescent secondary antibodies. Nuclei were counterstained with 4′,6-diamidino-2-phenylindole, and images were obtained using CLSM. Structural changes to the Golgi were also assessed by TEM of ultrathin sections. Enzymatic activities of Golgi-resident enzymes, Man II and GalT, were quantified using enzyme activity assay kits.

### PD-L1 glycosylation, surface expression, and exosome secretion

PD-L1 glycosylation in 4T1 cells was analyzed by Western blot, and *N*-glycopeptides were identified using nano LC–MS/MS. Membrane PD-L1 expression was quantified by flow cytometry after staining with PE-conjugated PD-L1 antibody. To analyze exosomal PD-L1, extracellular vesicles were isolated from treated cell supernatants using a standard differential centrifugation protocol. Exosome size was determined using a Flow NanoAnalyzer N30 E (NanoFCM), and exosomal protein markers were characterized by flow cytometry and TEM.

### *In vitro* ICD induction and bone marrow–derived dendritic cell maturation assay

To evaluate ICD, the release of damage-associated molecular patterns, including CRT, HMGB1, and ATP, was measured following treatment with various formulations containing 20 μg/ml OXA. CRT exposure was detected by immunofluorescence microscopy. HMGB1 localization was similarly assessed. ATP release was quantified using an ATP assay kit, whereas HMGB1 content in the supernatant was measured by ELISA.

For DC maturation assays, immature DCs were isolated from BALB/c mice and cocultured with pretreated 4T1 cells at a 1:1 ratio in 6-well plates. Maturation markers were analyzed by flow cytometry, and cytokine levels (IL-6, IFN-γ, and tumor necrosis factor-α) in the culture medium were determined using ELISA.

### LC–MS/MS analysis

#### LC–MS/MS analysis of *N*-glycopeptides

Proteins were extracted using radioimmunoprecipitation assay lysis buffer, and the supernatants were collected following centrifugation. For immunoprecipitation, beads were conjugated with the antibody for 2 h at room temperature and washed. Samples were then added to the antibody-conjugated beads, incubated for 2 h, and washed again before bead resuspension. Proteins were reduced with DTT (56 °C, 1 h), alkylated with iodoacetamide (room temperature, dark, 1 h), and digested overnight with trypsin at 37 °C. Peptides were desalted using a C18 tip and dried. Peptides were separated on a Vanquish Nano LC system equipped with a C18 column. A linear gradient from 4% to 95% mobile phase B was run over 131 min at 600 nl/min. Mass spectrometry (MS) analysis was performed on an Orbitrap Fusion Lumos Tribrid mass spectrometer. Full MS scans were acquired, followed by higher-energy collisional dissociation fragmentation of the top 20 precursors. Data were processed using MaxQuant (version 1.6.2.10, Max Planck Institute of Biochemistry, Martinsried, Germany) against a species-specific protein database.

### LC–MS/MS-based proteomics

#### Sample preparation and protein extraction

4T1 cells were lysed in 8 M urea buffer with 1 mM PMSF and 2 mM EDTA, sonicated, and centrifuged at 15,000*g* for 10 min at 4 °C. The protein concentration of supernatants was measured using a bicinchoninic acid assay.

#### Tryptic digestion and peptide desalting

Proteins were reduced with 10 mM DTT, alkylated with 50 mM iodoacetamide, and precipitated with cold acetone. The pellet was digested overnight with trypsin (Promega) in 25 mM ammonium bicarbonate at 37 °C. Peptides were desalted using C18 StageTips.

#### 4D-data-independent acquisition MS analysis

Peptides were separated on a nanoElute UHPLC (Bruker) with a 40-min acetonitrile gradient. Analysis was performed on a timsTOF Pro2 (Bruker) in data-independent acquisition–parallel accumulation serial fragmentation mode, covering ∗*m/z*∗ 400 to 1200 and ion mobility 0.85 to 1.3 Vs/cm^2^.

#### Data processing and protein identification

Data were analyzed using DIA-NN (version 1.8.1) against the UniProt *Mus musculus* database (UP000000589). The search included fixed carbamidomethylation and variable oxidation/N-terminal acetylation. The false discovery rate was set to 1% at both the peptide and protein levels. Protein quantification was performed using MaxLFQ.

#### Statistical and bioinformatics analysis

Differential protein abundance was assessed by a two-tailed *t* test (fold change [FC] ≥1.5 or ≤0.667, *p* < 0.05). Functional enrichment analysis was performed with clusterProfiler (v4.4.4). Protein–protein interaction networks were constructed using STRING.

### Biodistribution of OPCR-Lip

A 4T1 tumor model was established *via* subcutaneous injection of 5 × 10^6^ 4T1 cells suspended in PBS into the third mammary fat pad of BALB/c mice. Treatments commenced when tumors reached ∼100 mm^3^. To assess *in vivo* biodistribution, formulations labeled with DiD (5 μg/mouse) were administered *via* tail vein injection. Fluorescent imaging was performed at multiple time points. After the final imaging session, mice were sacrificed, and tumors and major organs were harvested for *ex vivo* fluorescence imaging.

### *In vivo* antitumor study

Female BALB/c mice were inoculated subcutaneously with 1 × 10^6^ 4T1 cells, and male C57BL/6J mice received the same number of B16F10 or LLC cells. Once tumors reached ∼100 mm^3^, mice were randomized into groups and treated with various formulations (OXA dose: 5 mg/kg), administered every other day for five doses. To assess immune activation, TDLNs, spleens, and tumors were harvested post-treatment. Tumors were dissociated into single-cell suspensions and stained with anti-CD11c, CD80, and CD86 antibodies for flow cytometry. TDLNs, spleens, and tumors were also stained with anti-CD3-EV450, CD8-ER780, and CD4-APC antibodies at 4 °C for 30 min to evaluate T-cell infiltration. For histological analysis, tumors and major organs (heart, liver, spleen, lung, and kidney) were fixed in 4% formaldehyde and subjected to H&E, Ki67, and TUNEL staining.

### Chronotherapy with OPCR-Lip

BALB/c mice were housed under a 12-h light/12-h dark cycle (lights on from 8:00 AM to 8:00 PM, corresponding to Zeitgeber time 0–12) with ad libitum access to food and water. Mice were subcutaneously inoculated with 1 × 10^6^ 4T1 cells. Once tumors reached ∼100 mm^3^, OPCR-Lip or PBS was administered at designated circadian time points for 2 weeks. Tumor weights were measured after euthanasia to evaluate time-dependent therapeutic efficacy.

### Bulk RNA-Seq and data processing

Breast tumors were isolated from female BALB/c mice; lung and skin tissues were collected from male C57BL/6J mice. Each tissue type had ≥2 biological replicates. Total RNA was extracted using TRIzol reagent (Invitrogen) per the manufacturer's instructions. Libraries were prepared and sequenced on an Illumina NovaSeq 6000 platform using 2 × 150 bp paired-end reads (PE150) by LC-Bio Technology. Transcript quantification was performed using Kallisto against the Ensembl v110 reference genome. Genes with transcripts per million >1 in any sample were considered expressed. Differential expression analysis was conducted using DESeq2, with genes showing |log_2_FC| >1 and a false discovery rate <0.05 considered significant. Pathway enrichment was performed using Metascape. Circadian gene analysis was conducted using MetaCycle, and enrichment analysis was performed using parametric gene set enrichment analysis.

### Single-nucleus RNA-Seq

#### Library preparation

Tissue samples were minced (∼1 mm fragments) and homogenized in 2 ml of ice-cold Nuclei EZ Lysis Buffer (NUC-101; Sigma–Aldrich) supplemented with protease (Roche) and RNase inhibitors (Promega, Life Technologies) using a Dounce homogenizer. After a 5-min incubation on ice, an additional 2 ml of lysis buffer was added. The sample was further homogenized and gently resuspended. After an additional 6 min on ice, 2 ml of 4% bovine serum albumin (BSA) was added to terminate lysis. The suspension was mixed, centrifuged (300*g*, 5 min, 4 °C), and resuspended in lysis buffer containing 4% BSA for 3 min. Debris was removed using Miltenyi Debris Removal Solution. The sample was washed with 4 ml buffer, incubated on ice (5 min), centrifuged again, and resuspended in Nuclei Suspension Buffer (1x PBS, 0.07% BSA, and 0.1% RNase inhibitor). The final nuclei suspension was filtered through a 20 μm cell strainer and quantified using a hemocytometer or Countess II Automated Cell Counter.

Single-nucleus suspensions (∼10,000 nuclei) were loaded onto the MobiNova-100 Single-Cell System using a MobiCube Single-Cell 3′ RNA-Seq Kit according to the manufacturer’s protocol. Complementary DNA amplification and library construction followed standard procedures. Sequencing was performed on an Illumina NovaSeq 6000 system (paired-end mode), targeting a minimum of 20,000 reads per nucleus, and conducted by LC-Bio Technology.

### Preprocessing, clustering, and annotation

Raw sequencing reads from snRNA-Seq were aligned to the GRCm39 reference genome using MobiVision software (v3.0), generating gene count matrices. A total of 35,790 nuclei were profiled across four libraries derived from four samples. Doublets were identified and removed using the scDblFinder R package (v1.16.0) (65). Nuclei were filtered based on three quality metrics: (i) expression of >300 genes with nonzero counts, (ii) >800 unique molecular identifiers, and (iii) mitochondrial gene content <5% of total reads. Genes expressed in fewer than 20 nuclei were excluded. After quality control, 19,744 genes across 32,206 nuclei remained. Adipocytes were further excluded, resulting in 32,087 high-quality nuclei for downstream analysis.

To integrate datasets across conditions, we used Seurat (v5.1.0) (66), normalizing and scaling each nucleus with the "SCTransform" function after regression on mitochondrial gene ratio, ribosomal gene ratio, and cell cycle scores. Batch effects were corrected using canonical correlation analysis on the top 2000 highly variable genes *via* the “IntegrateLayers” function. Principal component analysis was then applied, and the first 30 components were used to construct a shared nearest-neighbor graph *via* the “FindNeighbors” function. Clustering was performed using “FindClusters” at a resolution of 0.5. Dimensionality reduction was achieved with “RunUMAP” and visualized using UMAP.

Cell type annotation was based on canonical markers from previous tissue-specific studies. Marker genes for each cluster were identified using the Wilcoxon's rank-sum test *via* the "FindAllMarkers" function, selecting markers with avg_logFC >0.25, adjusted *p* < 0.05, and min.pct >0.20. T-cell subtypes were further resolved using established integration strategies.

### CNV analysis

To identify malignant cells, we used the inferCNV R package (v1.18.1). Raw, unintegrated count matrices were input, and only autosomal chromosomes were retained. Endothelial and mesenchymal stromal cells were used as reference cell types. Cells exhibiting substantial chromosomal copy number deviations were defined as malignant, displaying characteristic red-to-blue gradients on CNV heatmaps, representing gain or loss of genomic regions.

### Cell type abundance variation analysis

Cell type composition changes were assessed using the local true sign rate, which estimates the probability that the direction of the log-transformed FC is correct. A local true sign rate >0.9 was considered indicative of a statistically significant change in cell abundance between conditions.

### Pseudotime and potency score analysis

The differentiation trajectories of malignant cells before and after treatment were inferred using Monocle3 (v1.3.7) (67). Dimensionality reduction was performed for each malignant cell type, and trajectories were constructed in a semisupervised manner. Pseudotime values were calculated using the “pseudotime” function and discretized into integer bins. The proportion of treatment-group malignant cells within each bin was plotted against pseudotime, and a linear regression model was applied to compute *R*^2^ and *p* values.

Cellular potency scores were calculated using the “cytotrace2” function in CytoTRACE2, a deep learning framework that quantifies differentiation states based on single-cell transcriptomic profiles (33).

### Gene ontology analysis

Gene Ontology enrichment of DEGs was performed using Metascape (https://metascape.org/) (68).

### Statistical analysis

All statistical analyses were performed using SPSS (IBM SPSS Statistics 26). Data are reported as mean ± SD. For comparisons between two groups, unpaired Student’s *t* tests were used. For multigroup comparisons, one-way ANOVA followed by least significant difference post hoc tests (for normally distributed data) or Kruskal–Wallis tests with Dunn’s post hoc correction (for nonparametric data) were applied. Survival curves were analyzed using the log-rank (Mantel–Cox) test. A *p* value <0.05 was considered statistically significant.

## Data availability

All data are available in the main text or the supporting information. The MS data are available *via* ProteomeXchange (identifier: PXD073979). The reference genome and gene annotation file (GRCm39, Ensemble release 111) were downloaded from Ensembl (https://ftp.ensembl.org/pub/release-111/). All sequencing data are publicly available: bulk RNA-Seq of multiple tumor/normal tissues (PRJNA1304182), time-series bulk RNA-Seq of breast tumors (PRJNA1304155), and breast tumor snRNA-Seq (PRJNA1304156) *via* the National Center for Biotechnology Information Sequence Read Archive. The codes used in this study are available at https://github.com/RMIXXS/Mouse-breast-tumor-code.

## Supporting information

This article contains supporting information (69, 70, 71, 72, 73, 74, 75, 76, 77, 78, 79, 80, 81, 82, 83, 84, 85, 86, 87, 88, 89, 90, 91, 92, 93, 94, 95, 96, 97, 98).

## Conflict of interest

The authors declare that they have no conflicts of interest with the contents of this article.

## References

[1] Zhang Y., Zhang Z. (2020). The history and advances in cancer immunotherapy: understanding the characteristics of tumor-infiltrating immune cells and their therapeutic implications. Cell Mol. Immunol..

[2] Chen L., Han X. (2015). Anti-PD-1/PD-L1 therapy of human cancer: past, present, and future. J. Clin. Invest..

[3] Liu B., Song Y., Liu D. (2017). Recent development in clinical applications of PD-1 and PD-L1 antibodies for cancer immunotherapy. J. Hematol. Oncol..

[4] Lee T.A., Tsai E.Y., Liu S.H., Chou W.C., Hsu Hung S.D., Chang C.Y. (2025). Regulation of PD-L1 glycosylation and advances in cancer immunotherapy. Cancer Lett..

[5] Ding L., Guo H., Zhang J., Zheng M., Zhang W., Wang L. (2024). Zosuquidar promotes antitumor immunity by inducing autophagic degradation of PD-L1. Adv. Sci..

[6] Liu J., Qin J., Liang L., Zhang X., Gao J., Hao Y. (2024). Novel insights into the regulation of exosomal PD-L1 in cancer: from generation to clinical application. Eur. J. Pharmacol..

[7] Liu D.A., Tao K., Wu B., Yu Z., Szczepaniak M., Rames M. (2023). A phosphoinositide switch mediates exocyst recruitment to multivesicular endosomes for exosome secretion. Nat. Commun..

[8] Chen G., Huang A.C., Zhang W., Zhang G., Wu M., Xu W. (2018). Exosomal PD-L1 contributes to immunosuppression and is associated with anti-PD-1 response. Nature.

[9] Yang Y., Li C.W., Chan L.C., Wei Y., Hsu J.M., Xia W. (2018). Exosomal PD-L1 harbors active defense function to suppress T cell killing of breast cancer cells and promote tumor growth. Cell Res..

[10] Lee H.H., Wang Y.N., Xia W., Chen C.H., Rau K.M., Ye L. (2019). Removal of N-Linked glycosylation enhances PD-L1 detection and predicts Anti-PD-1/PD-L1 therapeutic efficacy. Cancer Cell.

[11] Xiong C., Jia L.N., Xiong W.X., Wu X.T., Xiong L.L., Wang T.H. (2023). Structural insights into substrate recognition and translocation of human peroxisomal ABC transporter ALDP. Signal Transduct. Target Ther..

[12] Majidpoor J., Mortezaee K. (2021). The efficacy of PD-1/PD-L1 blockade in cold cancers and future perspectives. Clin. Immunol..

[13] Wang Y.J., Fletcher R., Yu J., Zhang L. (2018). Immunogenic effects of chemotherapy-induced tumor cell death. Genes Dis..

[14] Chang H.N., Liu B.Y., Qi Y.K., Zhou Y., Chen Y.P., Pan K.M. (2015). Blocking of the PD-1/PD-L1 interaction by a D-Peptide antagonist for cancer immunotherapy. Angew. Chem. Int. Ed. Engl..

[15] Li H., Zhang P., Luo J., Hu D., Huang Y., Zhang Z.R. (2019). Chondroitin sulfate-linked prodrug nanoparticles target the Golgi apparatus for cancer metastasis treatment. ACS Nano.

[16] Tarragó-Trani M.T., Storrie B. (2007). Alternate routes for drug delivery to the cell interior: pathways to the Golgi apparatus and endoplasmic reticulum. Adv. Drug Deliv. Rev..

[17] Li Y., Chen Z.P., Xu D., Wang L., Cheng M.T., Zhou C.Z. (2024). Structural insights into Human ABCD3-mediated peroxisomal Acyl-CoA translocation. Cell Discov.

[18] Ye H., Wang K., Zhao J., Lu Q., Wang M., Sun B. (2023). In situ sprayed nanovaccine suppressing exosomal PD-L1 by Golgi apparatus disorganization for postsurgical melanoma immunotherapy. ACS Nano.

[19] Cervenkova L., Palek R., Moulisova V., Liska V., Daum O., Mohelnikova-Duchonova B. (2023). Protein expression and localization of ABC transporters in pancreatic adenocarcinoma: prognostic role of ABCC8. Pancreatology.

[20] Wang Y., Wang Y., Qin Z., Cai S., Yu L., Hu H. (2021). The role of non-coding RNAs in ABC transporters regulation and their clinical implications of multidrug resistance in cancer. Expert Opin. Drug Metab. Toxicol..

[21] Liu W.M., Nahar T.E.R., Jacobi R.H.J., Gijzen K., van Beek J., Hak E. (2012). Impaired production of TNF-α by dendritic cells of older adults leads to a lower CD8+ T cell response against influenza. Vaccine.

[22] Henry C.J., Ornelles D.A., Mitchell L.M., Brzoza-Lewis K.L., Hiltbold E.M. (2008). IL-12 produced by dendritic cells augments CD8^+^ T cell activation through the production of the chemokines CCL1 and CCL17. J. Immunol..

[23] Limagne E., Thibaudin M., Nuttin L., Spill A., Derangère V., Fumet J.D. (2019). Trifluridine/Tipiracil plus oxaliplatin improves PD-1 blockade in colorectal cancer by inducing immunogenic cell death and depleting macrophages. Cancer Immunol. Res..

[24] Vitali T., Sanchez-Alvarez R., Witkos T.M., Bantounas I., Cutiongco M.F.A., Dudek M. (2023). Vimentin intermediate filaments provide structural stability to the mammalian Golgi complex. J. Cell Sci..

[25] Appleman V.A., Matsuda A., Ganno M.L., Zhang D.M., Rosentrater E., Maldonado Lopez A.E. (2025). Selective STING activation in intratumoral myeloid cells via CCR2-directed antibody–drug conjugate TAK-500. Cancer Immunol. Res..

[26] Kulkarni A.M., Gayam P.K.R., Baby B.T., Aranjani J.M. (2025). Epithelial-mesenchymal transition in cancer: a focus on itraconazole, a hedgehog inhibitor. Biochim. Biophys. Acta Rev. Cancer.

[27] Babic A., Rosenthal M.H., Sundaresan T.K., Khalaf N., Lee V., Brais L.K. (2023). Adipose tissue and skeletal muscle wasting precede clinical diagnosis of pancreatic cancer. Nat. Commun..

[28] Sato K., Hikita H., Shigekawa M., Kato S., Sasaki Y., Shinkai K. (2022). Pentraxin 3 is an adipose tissue-related serum marker for pancreatic cancer cachexia predicting subsequent muscle mass and visceral fat loss. Cancer Sci..

[29] Bakhoum S.F., Cantley L.C. (2018). The multifaceted role of chromosomal instability in cancer and its microenvironment. Cell.

[30] Ma Y., Adjemian S., Mattarollo S.R., Yamazaki T., Aymeric L., Yang H. (2013). Anticancer chemotherapy-induced intratumoral recruitment and differentiation of antigen-presenting cells. Immunity.

[31] Lytle N.K., Barber A.G., Reya T. (2018). Stem cell fate in cancer growth, progression and therapy resistance. Nat. Rev. Cancer.

[32] Pattabiraman D.R., Bierie B., Kober K.I., Thiru P., Krall J.A., Zill C. (2016). Activation of PKA leads to mesenchymal-to-epithelial transition and loss of tumor-initiating ability. Sci. 2016.

[33] Kang M., Armenteros J.J.A., Gulati G.S., Gleyzer R., Avagyan S., Brown E.L. (2024). Mapping single-cell developmental potential in health and disease with interpretable deep learning. bioRxiv.

[34] Sharma P., Allison J.P. (2015). The future of immune checkpoint therapy. Science.

[35] Speiser D.E., Chijioke O., Schaeuble K., Münz C. (2023). CD4^+^ T cells in cancer. Nat. Cancer.

[36] Pauken K.E., Wherry E.J. (2015). SnapShot: t cell exhaustion. Cell.

[37] Wherry E.J., Kurachi M. (2015). Molecular and cellular insights into T cell exhaustion. Nat. Rev. Immunol..

[38] Ohdo S., Koyanagi S., Matsunaga N. (2023). Implications of biological clocks in pharmacology and pharmacokinetics of antitumor drugs. J. Contr. Release.

[39] Dong D., Yang D., Lin L., Wang S., Wu B. (2020). Circadian rhythm in pharmacokinetics and its relevance to chronotherapy. Biochem. Pharmacol..

[40] Sancar A., Van Gelder R.N. (2021). Clocks, cancer, and chronochemotherapy. Science.

[41] Wu G., Anafi R.C., Hughes M.E., Kornacker K., Hogenesch J.B. (2016). MetaCycle: an integrated R package to evaluate periodicity in large scale data. Bioinformatics.

[42] Deota S., Lin T., Chaix A., Williams A., Le H., Calligaro H. (2023). Diurnal transcriptome landscape of a multi-tissue response to time-restricted feeding in mammals. Cell Metab..

[43] Yang Y., Abdo A.N., Kawara H., Selby C.P., Sancar A. (2023). Preservation of circadian rhythm in hepatocellular cancer. J. Biol. Chem..

[44] Hassan S.A., Ali A.A.H., Yassine M., Sohn D., Pfeffer M., Jänicke R.U. (2021). Relationship between locomotor activity rhythm and corticosterone levels during HCC development, progression, and treatment in a mouse model. J. Pineal. Res..

[45] Yang Y., Adebali O., Wu G., Selby C.P., Chiou Y.Y., Rashid N. (2018). Cisplatin-DNA adduct repair of transcribed genes is controlled by two circadian programs in mouse tissues. Proc. Natl. Acad. Sci. U. S. A..

[46] Kang T.H., Lindsey-Boltz L.A., Reardon J.T., Sancar A. (2010). Circadian control of XPA and excision repair of cisplatin-DNA damage by cryptochrome and HERC2 ubiquitin ligase. Proc. Natl. Acad. Sci. U. S. A..

[47] Yang Y., Liu Z., Selby C.P., Sancar A. (2019). Long-term, genome-wide kinetic analysis of the effect of the circadian clock and transcription on the repair of cisplatin-DNA adducts in the mouse liver. J. Biol. Chem..

[48] Wang Z., Li F., Wei M., Zhang S., Wang T. (2020). Circadian clock protein period2 suppresses the pi3k/akt pathway and promotes cisplatin sensitivity in ovarian cancer. Cancer Manag. Res..

[49] Katamune C., Koyanagi S., ichi Hashikawa K., Kusunose N., Akamine T., Matsunaga N. (2019). Mutation of the gene encoding the circadian clock component PERIOD2 in oncogenic cells confers chemoresistance by up-regulating the Aldh3a1 gene. J. Biol. Chem..

[50] Tang Q., Xie M., Yu S., Zhou X., Xie Y., Chen G. (2019). Periodic oxaliplatin administration in synergy with PER2-mediated PCNA transcription repression promotes chronochemotherapeutic efficacy of OSCC. Adv. Sci..

[51] Ortiz-Tudela E., Mteyrek A., Ballesta A., Innominato P.F., Levi F. (2013). Cancer chronotherapeutics: experimental, theoretical, and clinical aspects. Handb. Exp. Pharmacol..

[52] Dallmann R., Okyar A., Lévi F. (2016). Dosing-time makes the poison: circadian regulation and pharmacotherapy. Trends Mol. Med..

[53] Nagato T., Lee Y.R., Harabuchi Y., Celis E. (2014). Combinatorial immunotherapy of polyinosinic-polycytidylic acid and blockade of programmed death-ligand 1 induce effective CD8 t-cell responses against established tumors. Clin. Cancer Res..

[54] Zaremba-Czogalla M., Hryniewicz-Jankowska A., Tabola R., Nienartowicz M., Stach K., Wierzbicki J. (2018). A novel regulatory function of CDKN1A/p21 in TNFα-induced matrix metalloproteinase 9-dependent migration and invasion of triple-negative breast cancer cells. Cell. Signal..

[55] Ming D., Ma J. (2022). EphA3 targeted by miR-3666 contributes to melanoma malignancy via activating ERK1/2 and p38 MAPK pathways. Open Med..

[56] Li X., Chen S., Shi Y., Wang Y., Wang X., Lin Q. (2024). Transcription factor MEF2D regulates aberrant expression of ACSL3 and enhances sorafenib resistance by inhibiting ferroptosis in HCC. Front. Pharmacol..

[57] Noujarède J., Carrié L., Garcia V., Grimont M., Eberhardt A., Mucher E. (2023). Sphingolipid paracrine signaling impairs keratinocyte adhesion to promote melanoma invasion. Cell Rep.

[58] Fico F., Santamaria-Martínez A. (2020). TGFBI modulates tumour hypoxia and promotes breast cancer metastasis. Mol. Oncol..

[59] Pai J.T., Hsu C.Y., Hsieh Y.S., Tsai T.Y., Hua K.T., Weng M.S. (2020). Suppressing migration and invasion of H1299 lung cancer cells by honokiol through disrupting expression of an HDAC6-mediated matrix metalloproteinase 9. Food Sci. Nutr..

[60] Zhang X., Zhu S., Luo G., Zheng L., Wei J., Zhu J. (2007). Expression of MMP-10 in lung cancer. Anticancer Res..

[61] Pang K., Shi Z., Wei L., Dong Y., Ma Y., Wang W. (2023). Research progress of therapeutic effects and drug resistance of immunotherapy based on PD-1/PD-L1 blockade. Drug Resist. Updat..

[62] Wang X.D., Wua M., Zhang B.M., Zhang Y.L., Hua Z.X., Shi L. (2019). Phase-transfer method synthesis hydroxyethyl cellulose lauryl ether. Colloid Surf. A.

[63] Karaki N., Aljawish A., Humeau C., Muniglia L., Jasniewski J. (2016). Enzymatic modification of polysaccharides: mechanisms, properties, and potential applications: a review. Enzyme Microb. Technol..

[64] Yao Q., Kou L., Tu Y., Zhu L. (2018). MMP-responsive 'smart' drug delivery and tumor targeting. Trends Pharmacol. Sci..

[65] Germain P.L., Lun A., Macnair W., Robinson M.D., Robinson M.D. (2021). Doublet identification in single-cell sequencing data using scDblFinder. F1000Res.

[66] Hao Y., Stuart T., Kowalski M., Choudhary S., Hoffman P., Hartman A. (2024). Dictionary learning for integrative, multimodal, and scalable single-cell analysis. Nat. Biotechnol..

[67] Cao J., Spielmann M., Qiu X., Huang X., Ibrahim D.M., Hill A.J. (2019). The single-cell transcriptional landscape of Mammalian organogenesis. Nature.

[68] Zhou Y., Zhou B., Pache L., Chang M., Khodabakhshi A.H., Tanaseichuk O. (2019). Metascape provides a biologist-oriented resource for the analysis of systems-level datasets. Nat. Commun..

[69] Wei Y., Bao R., Hu L., Geng Y., Chen X., Wen Y. (2023). Ti3C2 (MXene) nanosheets disrupt spermatogenesis in male mice mediated by the ATM/p53 signaling pathway. Biol. Direct..

[70] Genc S., Cicek B., Yeni Y., Kuzucu M., Hacimuftuoglu A., Bolat I. (2024). Morinda citrifolia protective effects on paclitaxel-induced testis parenchyma toxicity: an experimental study. Reprod. Toxicol..

[71] Huang Y., Jiao Z., Fu Y., Hou Y., Sun J., Hu F. (2024). An overview of the functions of p53 and drugs acting either on wild- or mutant-type p53. Eur. J. Med. Chem..

[72] Zhang H., Xu J., Long Y., Maimaitijiang A., Su Z., Li W., Li J. (2024). Unraveling the guardian: p53’s multifaceted role in the DNA damage response and tumor treatment strategies. Int. J. Mol. Sci..

[73] Hou T., Cao Z., Zhang J., Tang M., Tian Y., Li Y. (2020). SIRT6 coordinates with CHD4 to promote chromatin relaxation and DNA repair. Nucleic Acids Res..

[74] Huang S., Yan Q., Xiong S., Peng Y., Zhao R., Liu C. (2020). Chromodomain helicase DNA-binding protein 5 inhibits renal cell carcinoma tumorigenesis by activation of the p53 and RB pathways. Biomed. Res. Int..

[75] Chandramouly G. (2022). Gadd45 in DNA demethylation and DNA repair. Adv. Exp. Med. Biol..

[76] Humayun A., Fornace A.J. (2022). GADD45 in stress signaling, cell cycle control, and apoptosis. Adv. Exp. Med. Biol..

[77] Wang K.Y., Wang K.J., Shen L.L., Wang X.H. (2024). The down-regulation of GADD45B leads to a conversion of cellular oxidative phosphorylation to glycolysis and promotes the progression of bladder cancer. Heliyon.

[78] Youn C.K., Lee J.H., Hariharasudhan G., Kim H.B., Kim J., Lee S. (2022). HspBP1 is a dual function regulatory protein that controls both DNA repair and apoptosis in breast cancer cells. Cell Death Dis..

[79] Dubrez L., Causse S., Borges Bonan N., Dumétier B., Garrido C. (2020). Heat-shock proteins: chaperoning DNA repair. Oncogene.

[80] Szczepny A., Carey K., McKenzie L., Jayasekara W.S.N., Rossello F., Gonzalez-Rajal A. (2018). The Tumor suppressor Hic1 maintains chromosomal stability independent of Tp53. Oncogene.

[81] Zhao T., Yang T., Zhang J., Hao H., Wang D. (2018). The tumor suppressor Hic1 maintains chromosomal stability independent of Tp53. Gene.

[82] Chen Y., Jiao D., Liu Y., Xu X., Wang Y., Luo X. (2023). FBXL4 mutations cause excessive mitophagy via BNIP3/BNIP3L accumulation leading to mitochondrial DNA depletion syndrome. Cell Death Differ..

[83] Fu L.H., Qi C., Sun T., Huang K., Lin J., Huang P. (2023). Glucose oxidase-instructed biomineralization of calcium-based biomaterials for biomedical applications. Exploration.

[84] Song Z., Feng Z., Wang X., Li J., Zhang D. (2025). NFKB1 as a key player in tumor biology: from mechanisms to therapeutic implications. Cell Biol. Toxicol..

[85] Pol J.G., Lizarralde-Guerrero M., Kroemer G. (2024). Immunogenic oncolysis by tigilanol tiglate. OncoImmunology.

[86] Rahman S.M.T., Singh A., Lowe S., Aqdas M., Jiang K., Vaidehi Narayanan H. (2024). Co-imaging of RelA and c-Rel reveals features of NF-κB signaling for ligand discrimination. Cell Rep.

[87] Zhang Y.J., Huang C., Zu X.G., Liu J.M., Li Y.J. (2024). Use of machine learning for the identification and validation of immunogenic cell death biomarkers and immunophenotypes in coronary artery disease. J. Inflamm. Res..

[88] Li Y.J., Geng W.L., Li C.C., Wu J.H., Gao F., Wang Y. (2024). Progress of CCL20-CCR6 in the airways: a promising new therapeutic target. J. Inflamm..

[89] Shen M., Cao S., Long X., Xiao L., Yang L., Zhang P. (2024). DNAJC12 causes breast cancer chemotherapy resistance by repressing doxorubicin-induced ferroptosis and apoptosis via activation of AKT. Redox Biol..

[90] Ochoa D., Hercules A., Carmona M., Suveges D., Gonzalez-Uriarte A., Malangone C. (2021). Open targets platform: supporting systematic drug-target identification and prioritisation. Nucleic Acids Res..

[91] Gedik M.E., Saatci O., Oberholtzer N., Uner M., Caliskan O.A., Cetin M. (2024). Targeting TACC3 induces immunogenic cell death and enhances T-DM1 response in HER2-positive breast cancer. Cancer Res..

[92] Li J.J., Mao J.X., Zhong H.X., Zhao Y.Y., Teng F., Lu X.Y. (2024). Multifaceted roles of lymphatic and blood endothelial cells in the tumor microenvironment of hepatocellular carcinoma: a comprehensive review. World J. Hepatol..

[93] Krause S.W., Kehli M., Andreesen R., Rehli M., Ylndreesen R. (1998). Carboxypeptidase M as a marker of macrophage maturation Autbors’ addresses immunological reviews. Immunol. Rev..

[94] Song J., Liu L., Wang Z., Xie D., Azami N.L.B., Lu L. (2024). CCL20 and CD8A as potential diagnostic biomarkers for HBV-induced liver fibrosis in chronic hepatitis B. Heliyon.

[95] Maroui M.A., Odongo G.A., Mundo L., Manara F., Mure F., Fusil F. (2024). Aflatoxin B1 and Epstein–Barr virus-induced CCL22 expression stimulates B cell infection. Proc. Natl. Acad. Sci. U. S. A..

[96] Guo C., Dai X., Du Y., Xiong X., Gui X. (2024). Preclinical development of a novel CCR8/CTLA-4 bispecific antibody for cancer treatment by disrupting CTLA-4 signaling on CD8 T cells and specifically depleting tumor-resident Tregs. Cancer Immunol. Immunother..

[97] Glaviano A., Lau H.S.H., Carter L.M., Lee E.H.C., Lam H.Y., Okina E. (2025). Harnessing the tumor microenvironment: targeted cancer therapies through modulation of epithelial-mesenchymal transition. J. Hematol. Oncol..

[98] Blomberg K.E.M., Boucheron N., Lindvall J.M., Yu L., Raberger J., Berglöf A. (2009). Transcriptional signatures of Itk-Deficient CD3+, CD4+ and CD8+ T-Cells. BMC Genomics.

