# Supplementary material for: Targeting the Golgi apparatus enhances PD-L1 blockade and synergizes with oxaliplatin to improve immunotherapy efficacy
Source: J Biol Chem. 2026 Mar 4;302(4):111343. doi: 10.1016/j.jbc.2026.111343 (PMC13053745; doi:10.1016/j.jbc.2026.111343)
Supplement: Supplementary Material [file mmc1.docx]

**Supporting information**

Targeting the Golgi apparatus enhances PD-L1 blockade and synergizes with oxaliplatin to improve immunotherapy efficacy

Haohuan Li,^1#*^ Chao Cui, ^1#^ Chenglu Sun,^1#^ Ziyu Chen,^2#^ Dengfeng Gao,^2^ Peng Yuan,^1^ Shibo Tian,^1^ Qin Zhong,^1^ Funeng Xu,^1^ Xiaoxia Liang,^1^ Long Jin,^2^ Keren Long,^2^ Lu Lu,^2^ Juan Deng,^2^ Jiaxue Cao,^2^ Xiaolan Fan,^2^ Fanli Kong,^2^ Chengdong Wang,^3^ Desheng Li,^3^ Zhiyong Qian,^4*^ Mingzhou Li ^2,5*^

1. College of Veterinary Medicine, Sichuan Agricultural University, Chengdu, Sichuan 611130, China.

2. College of Animal Science and Technology, Sichuan Agricultural University, Chengdu, Sichuan 611130, China.

3. China Conservation and Research Centre for the Giant Panda, Chengdu, Sichuan 611830, China

4. Department of Biotherapy, Cancer Center and State Key Laboratory of Biotherapy, West China Hospital, Sichuan University, Chengdu, Sichuan 610041, China

5. College of Life Science, China West Normal University, Nanchong, 637009, Sichuan, China

﻿# These authors have contributed equally to this work

﻿*Correspondence

﻿Prof. Mingzhou Li, Prof. Zhiyong Qian, Dr Haohuan Li

Email: mingzhou.li@sicau.edu.cn; zhiyongqian@scu.edu.cn; lihaohuan@sicau.edu.cn

**Supplementary Figures**

**
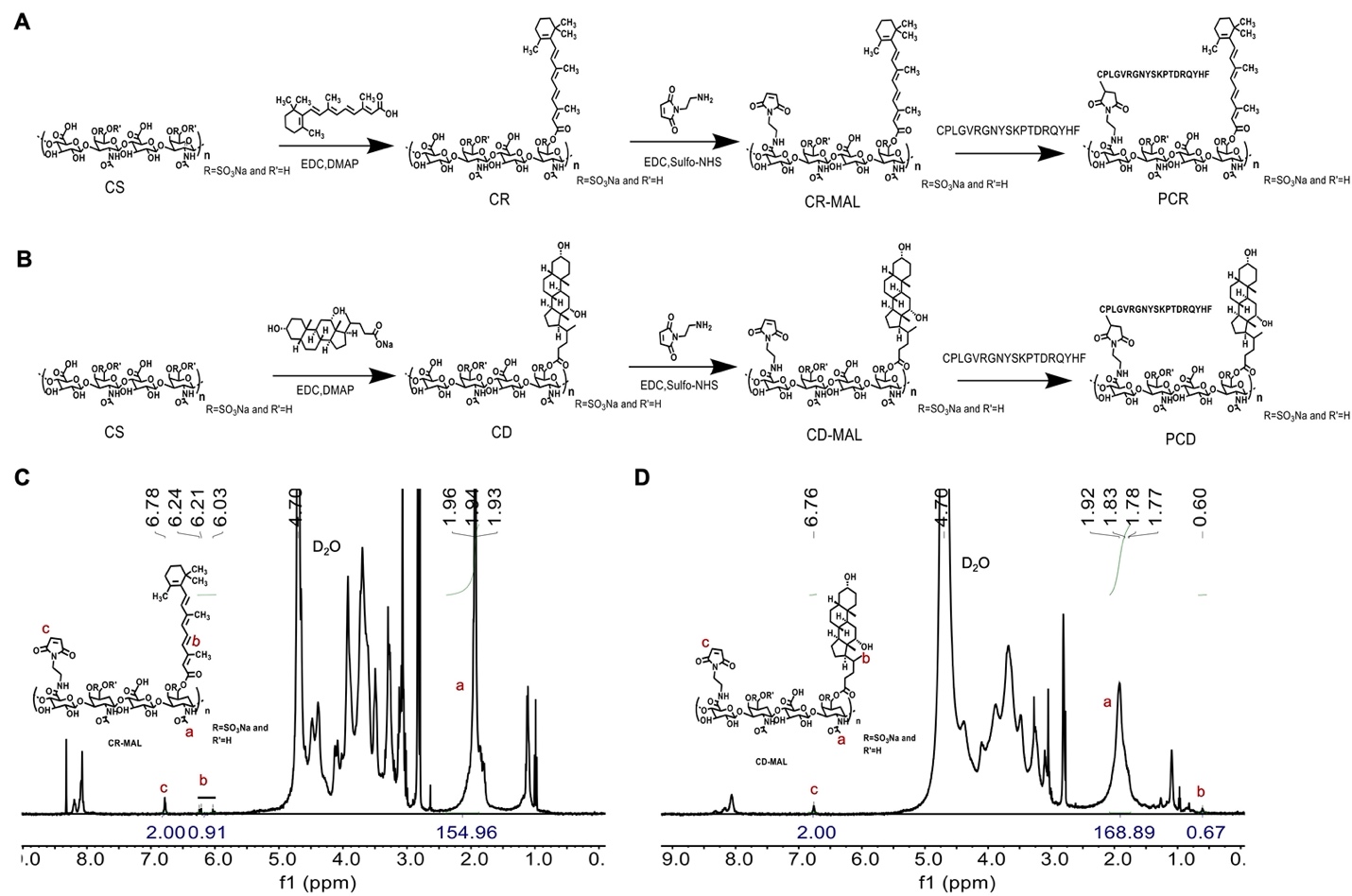
**

**Figure S1. Characterization of PCR-Lip**. ﻿*A* and *B*, Synthetic schemes of PCR and PCD.  *C* and *D*, ^1^H NMR spectra of CR-MAL and CD-MAL. The coupling efficiencies were 1.36% for RA (determined by HPLC) and 1.96% for MAL (determined by ¹H NMR).


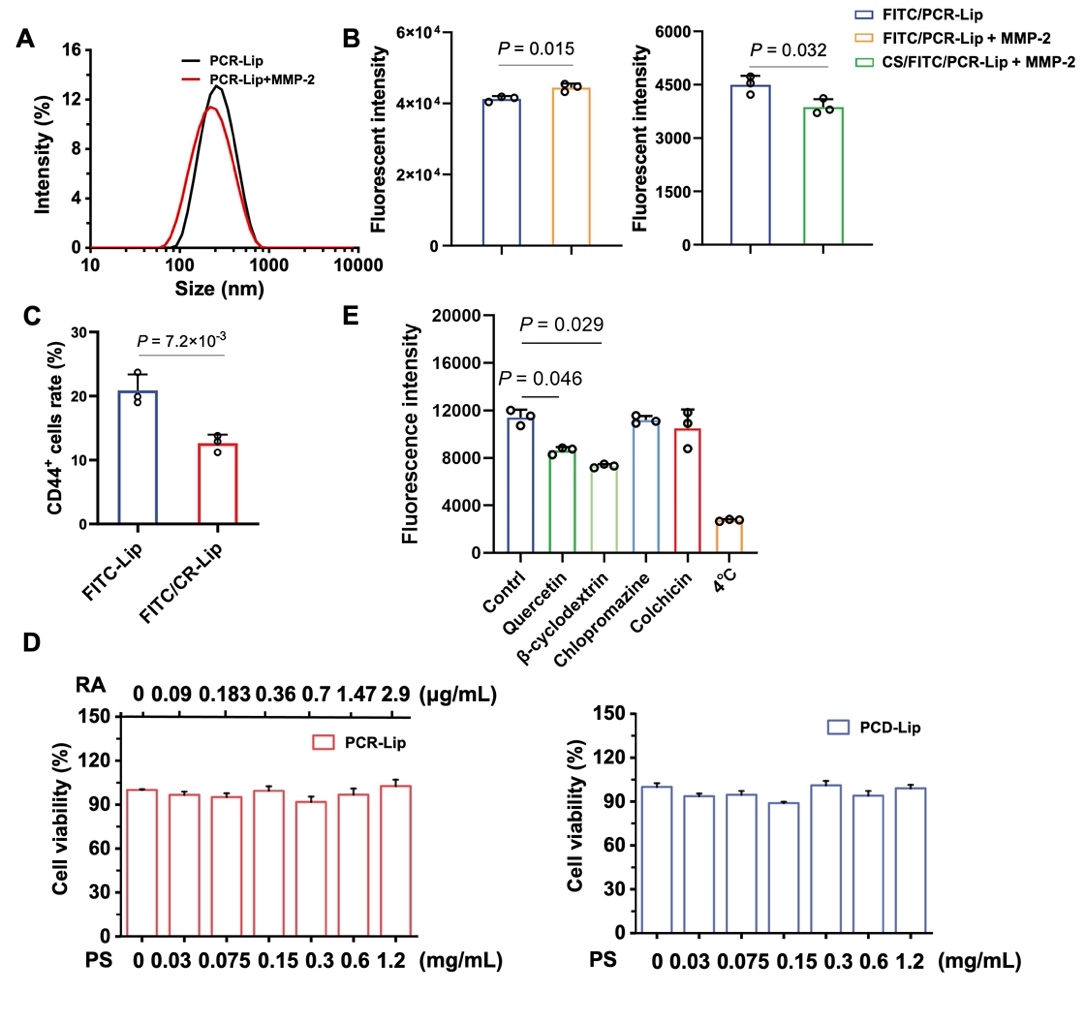


**Figure S2. Cellular uptake and MMP-2 sensitivity of PCR-Lip.** *A*, Size distribution of PCR-Lip after 3 hours incubation with 50 µg/mL MMP-2 at 37 °C. *B,* Flow cytometry analysis of mean fluorescence intensity (MFI) in 4T1 cells. Cells were either pre‑treated with chondroitin sulfate (CS) to block CD44 receptors, followed by incubation with FITC‑labeled PCR‑Lip and MMP‑2 (2.5 µg/mL) for 4 hours (“CS + FITC/PCR‑Lip + MMP‑2”), or directly incubated with FITC‑labeled PCR‑Lip and MMP‑2 (“FITC/PCR‑Lip + MMP‑2”) under the same conditions (*n* = 3). ﻿C, Flow cytometry analysis of anti-CD44 antibody binding to the surface of 4T1 cells following a 1 hour incubation with either FITC labeled liposomes (FITC-Lip) or FITC-labeled CS-modified liposomes (FITC/CR-Lip) (*n* = 3). *D,* ﻿Viability of 4T1 cells treated with various concentrations of PCR–Lip and PCD-Lip for 24 hours, PCR-Lip concentrations are quantified by phospholipid (PS) content, with corresponding retinoic acid (RA) concentrations (*n* = 3). *E,* Quantification of MFI from flow cytometry following 4 hours of incubation of FITC/PCR-Lip with 4T1 cells in the presence of different uptake inhibitors (*n* = 3). Data are shown as mean ± SD.


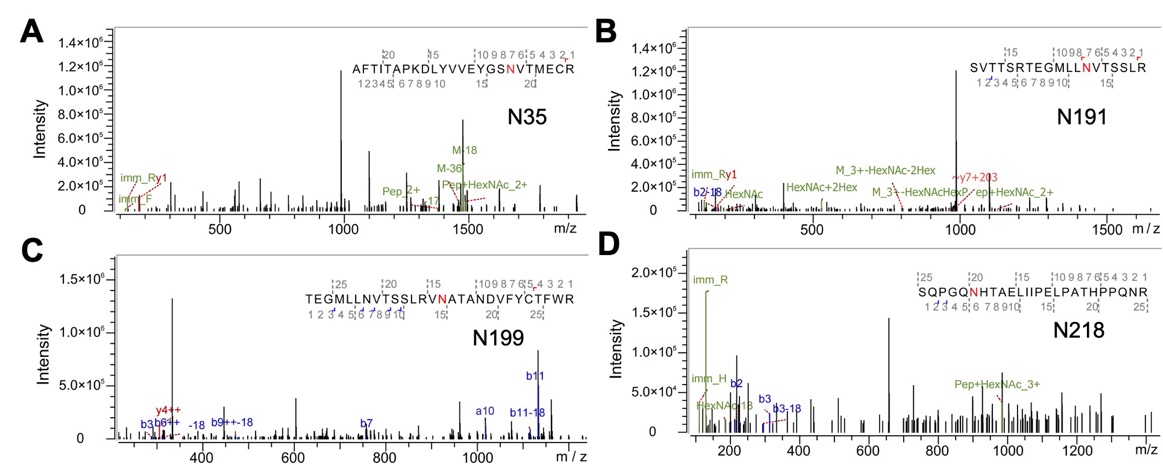
**Figure S3. LC-MS/MS analysis of N-glycopeptides**. *A-D,* LC-MS/MS spectra of N-glycopeptides identified in 4T1 cells at *N*-glycosylation sites N35, N191, N199, and N218.


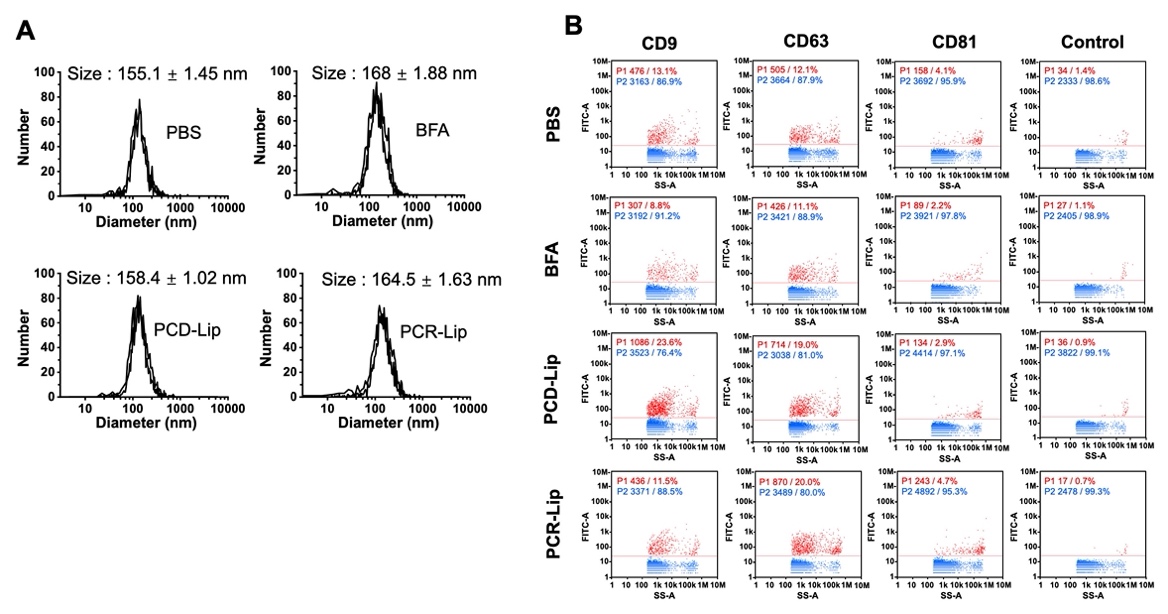
**Figure S4. Exosome identification from 4T1 cells.** *A,* Size distribution of exosomes isolated from PCR-Lip–treated 4T1 cells, measured by nanoparticle tracking analysis. *B*, Flow cytometry analysis of exosomal markers CD9, CD63, and CD81 in samples treated with different formulations.


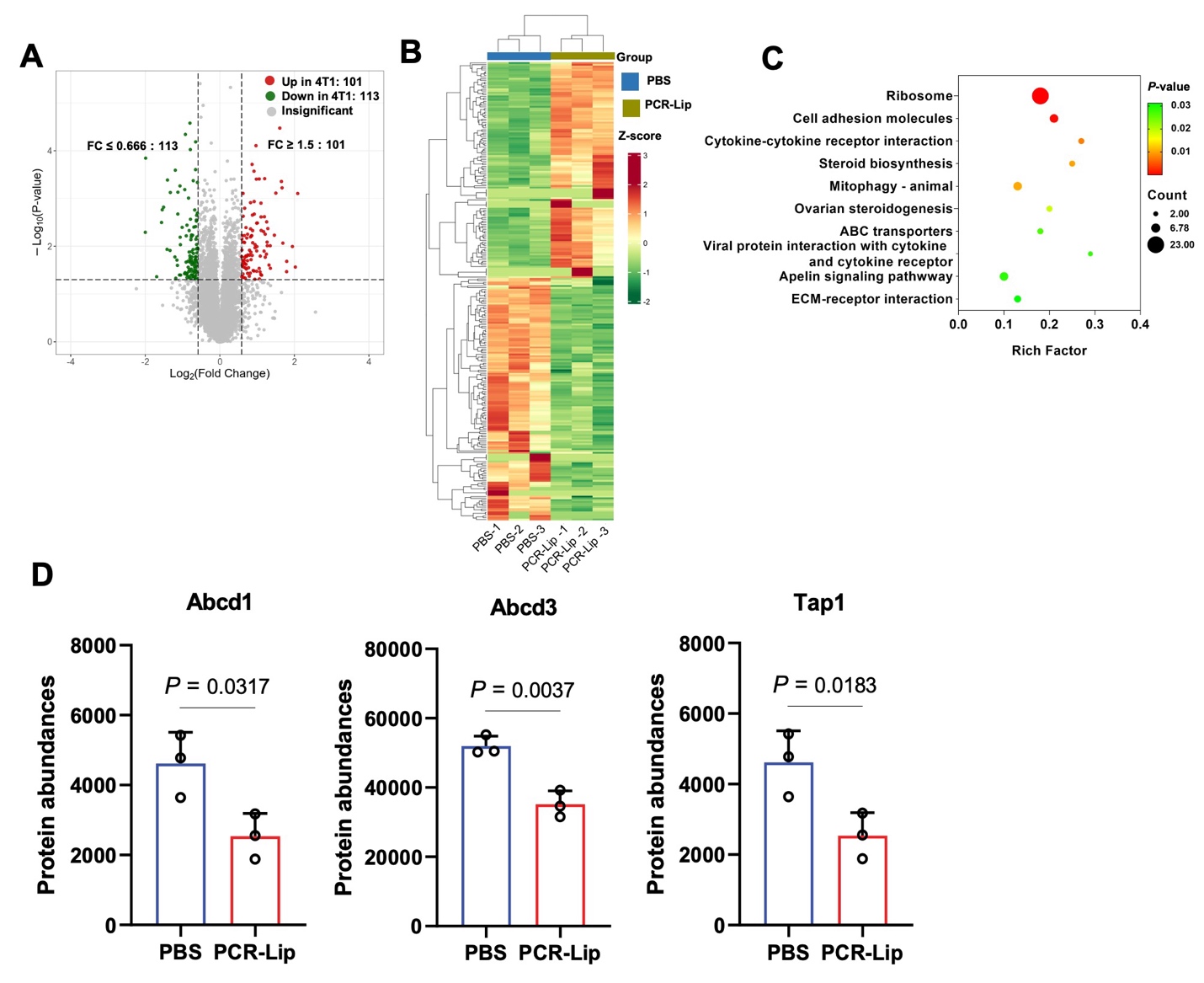


**Figure S5. Differential abundance analysis of proteins induced by PCR-Lip.** *A,* Volcano plot of differential abundance analysis between proteomes of 4T1 cells treated with PCR-Lip versus PBS (*n* = 3). Each dot represents a single protein. Proteins with log_2_ fold change (FC) ≥ 1.5, *P* value ≤ 0.05 and log_2_ FC ≤ 0.666, *P* value ≤ 0.05 are shown as red and green dots, respectively. *B,* Heatmap showing the relative abundance (Z-score) of proteins that are significantly different in 4T1 cells between the PBS and PCR-Lip groups. *C,* KEGG pathway enrichment analysis of differentially expressed proteins in 4T1 cells treated with PCR-Lip compared to those treated with PBS. *D,* Protein levels of ABC transporter-associated markers (Abcd1, Abcd3, Tap1) in 4T1 cells post-treatment. Data are presented as means ± SD.


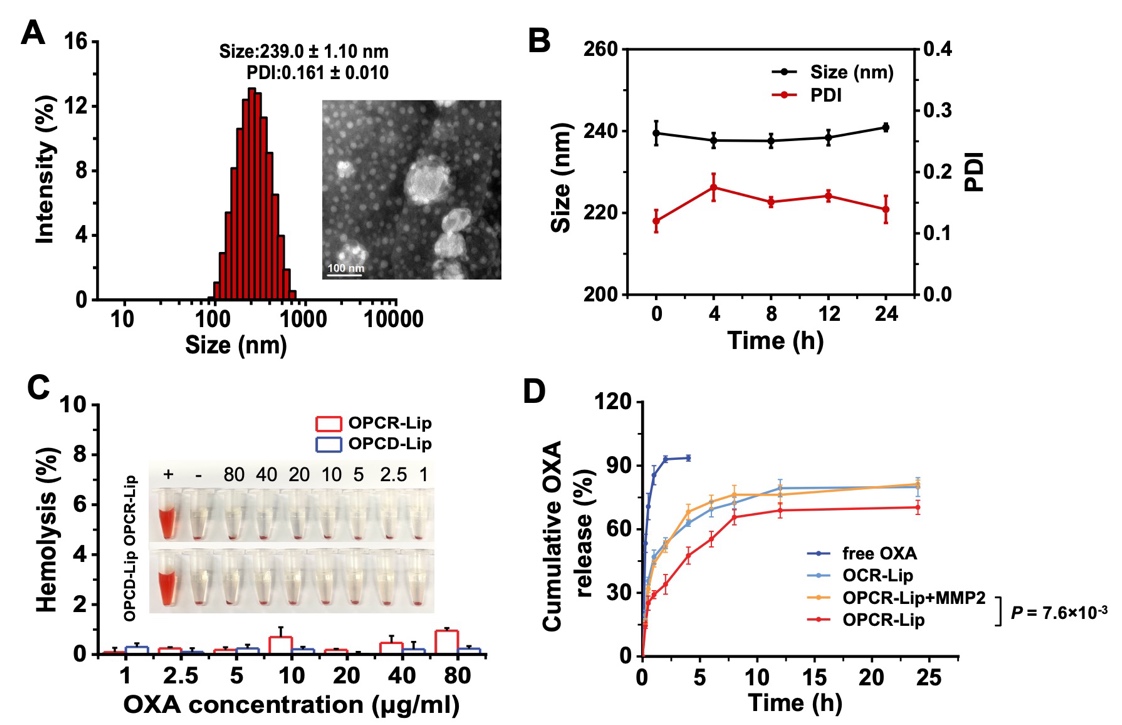


**Figure S6. Characterization of OPCR-Lip.** *A* and *B*, TEM image and size distribution, and colloidal stability in 50% serum of OPCR-Lip (*n* = 3). *C,* Hemolytic activity of OPCR-Lip at varying concentrations (*n* = 3). *D,* *In vitro* release profile of OXA from OPCR-Lip (*n* = 3). Data are presented as mean ± SD.


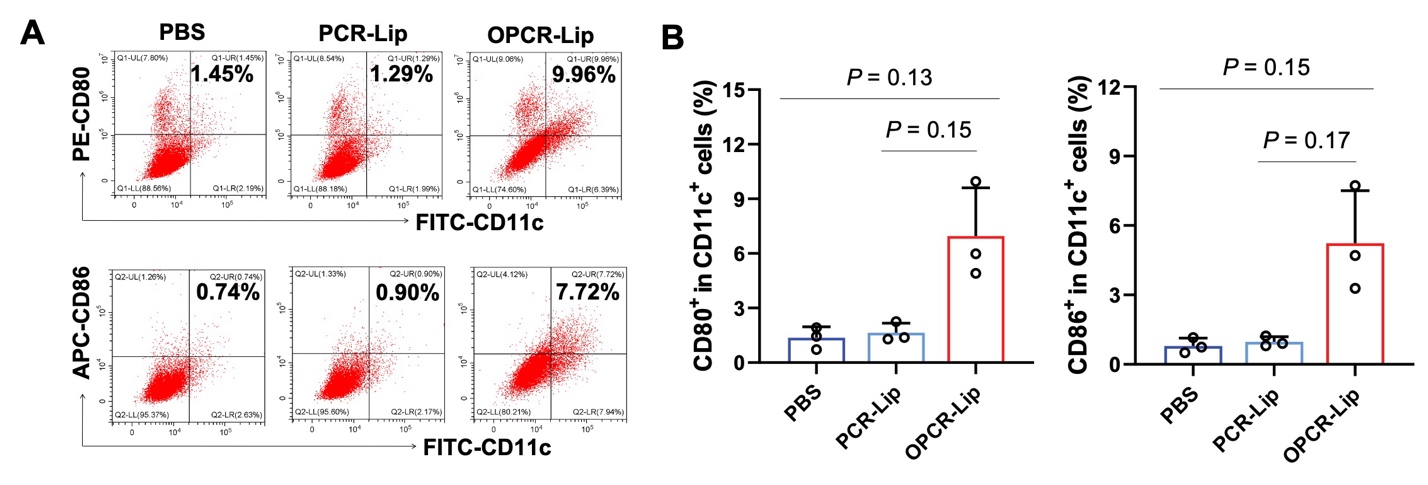
**Figure S7. *In vivo* activation of dendritic cells (DCs) by OPCR-Lip.** *A,* Representative flow cytometry plots. *B,* Quantitative analysis of mature DCs in tumor-draining lymph nodes from 4T1 tumor-bearing mice following treatment with different formulations (*n* = 3).


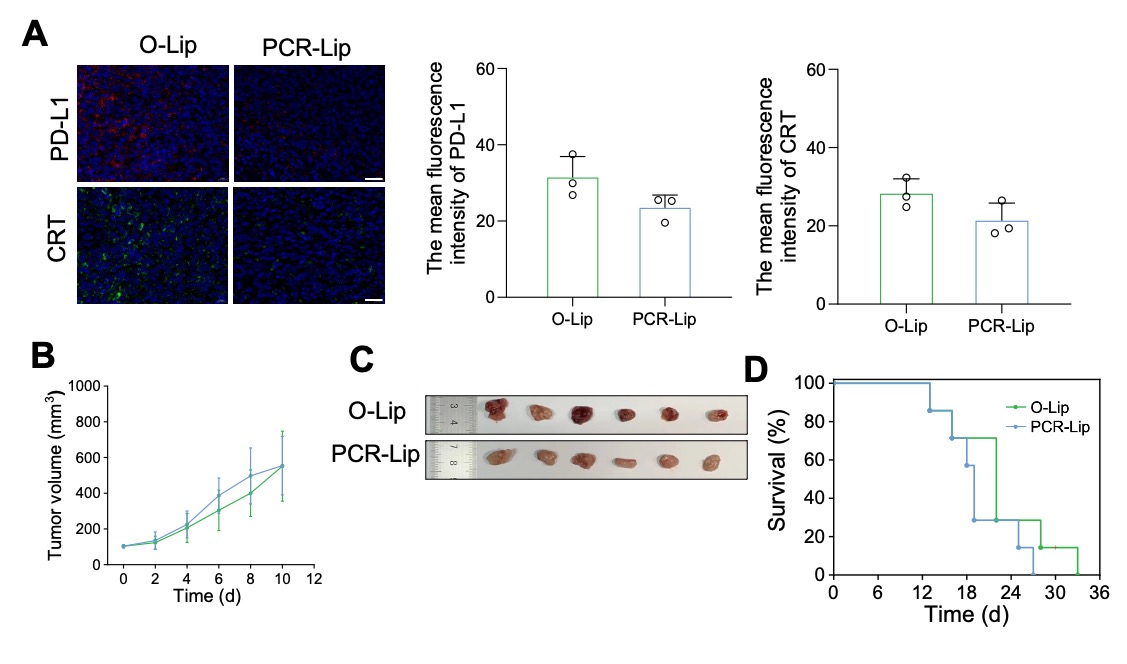


**Figure S8. *In vivo* antitumor efficacy of O-Lip and PCR-Lip.** *A*, Representative immunofluorescence images showing PD-L1 and CRT expression in tumor sections post-treatment. Scale bar: 100 μm. *B,* Tumor growth curves of 4T1 tumor-bearing mice treated with various formulations via intravenous injection (*n* = 6). *C,* Representative images of tumors excised from each treatment group. Solid black lines separate the groups, and a ruler (mm) indicates scale. *D,* Kaplan–Meier survival curves of 4T1 tumor-bearing mice following different treatments (*n* = 6). All data are presented as mean ± SD.


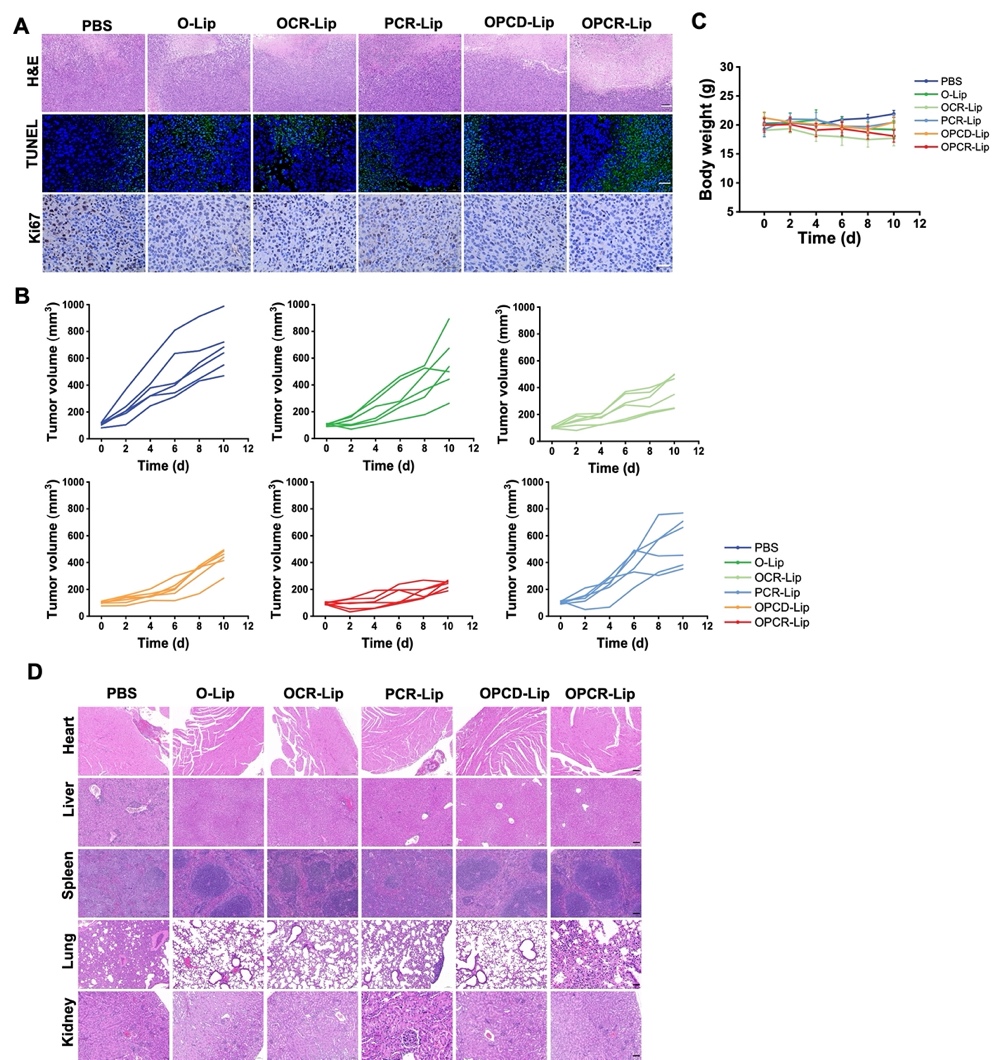


**Figure S9. *In vivo* antitumor efficacy.** *A,* H&E, TUNEL, and Ki67 staining of tumor sections from treated mice. Scale bar: 100 μm, 50 μm and 40 μm. *B,* Tumor growth curves for individual 4T1-bearing mice following intravenous administration of different formulations (*n* = 6). *C,* Body weight changes over time (*n* = 6). *D,* H&E staining of major organs from treated mice. Scale bar: 100 μm.


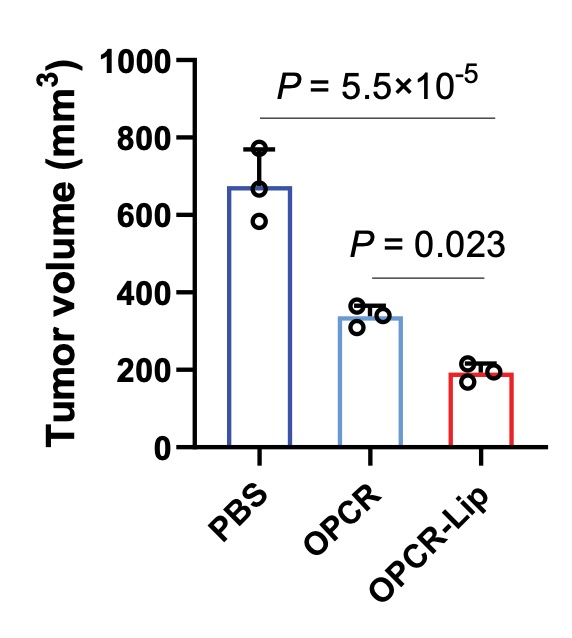


**﻿Figure S10.** Tumor volume in 4T1 tumor-bearing mice after treatment with various formulations via intravenous injection (*n* = 3).

**
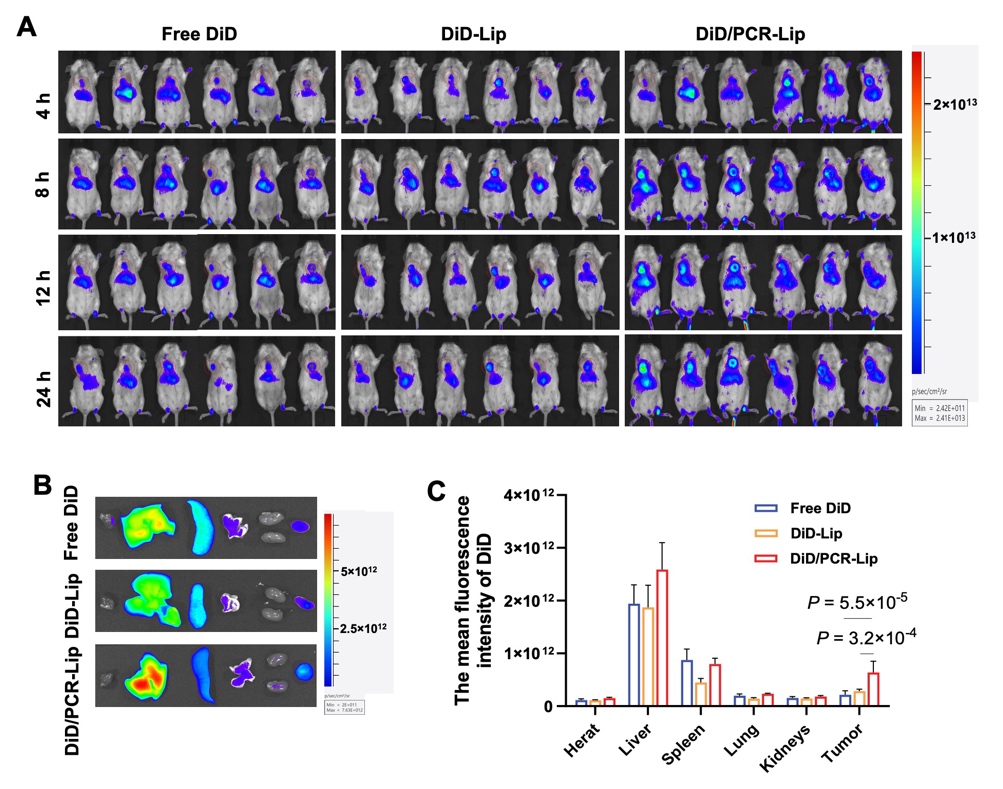
Figure S11. Biodistribution of OPCR-Lip *in vivo.*** *A,* *In vivo* fluorescence imaging of tumors following intravenous injection of free DiD, DiD-Lip, or DiD/PCR-Lip at various time points. *B, Ex vivo* fluorescence images of isolated organs and tumors 24 hours post-injection. *C,* Semi-quantitative analysis of mean fluorescence intensity (*n* = 6).

**
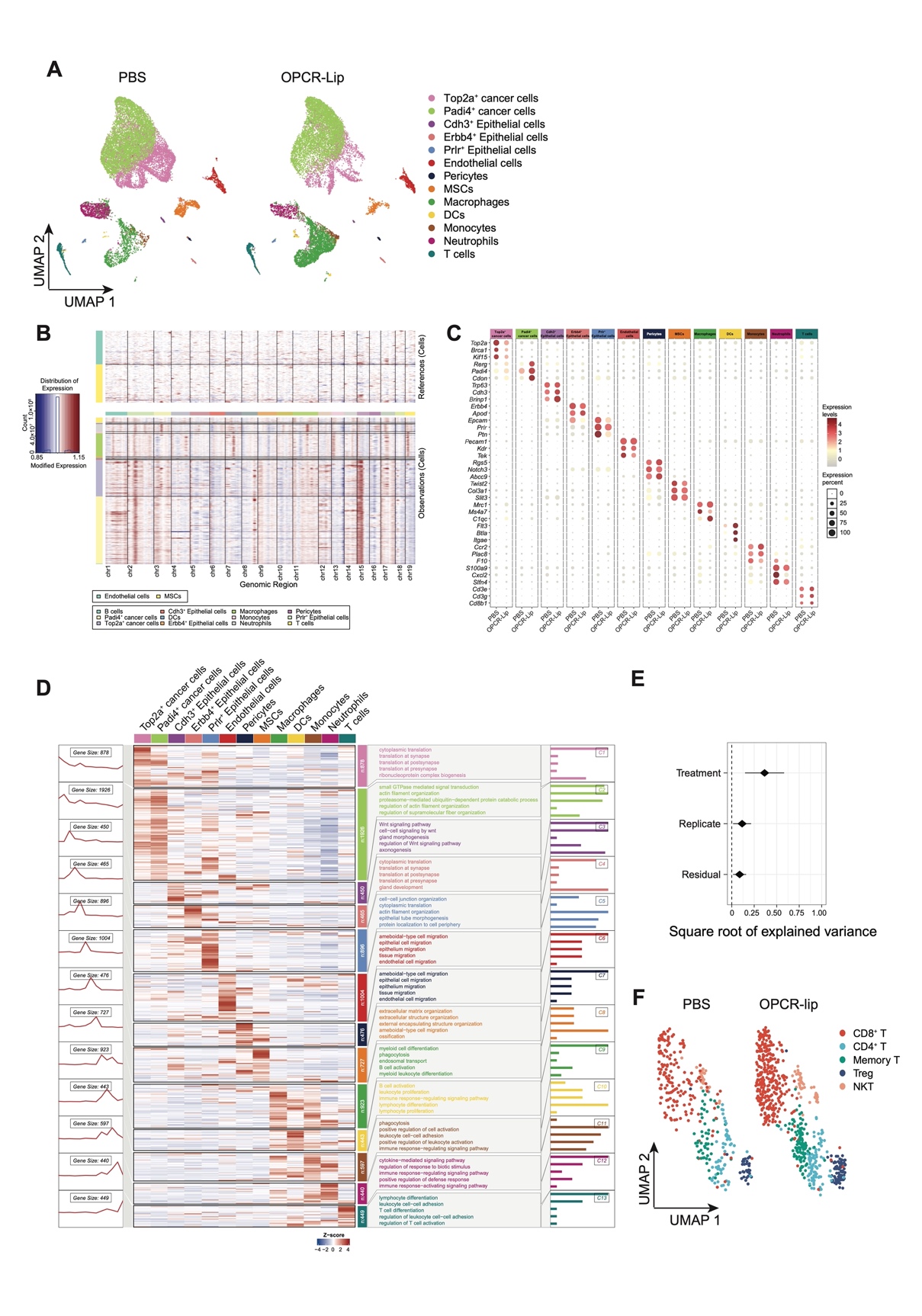
**

**Figure S12. Single-nucleus transcriptomic analysis of tumor tissues from OPCR-Lip–treated mice.** *A,* UMAP projection of identified cell clusters. *B,* Copy number variation (CNV) heatmap across clusters, with mesenchymal stromal cells (MSCs) and endothelial cells as controls. Red indicates genomic gains; blue indicates losses. *C,* Dot plot showing expression of canonical marker genes across clusters in snRNA-seq data. *D,* Enrichment analysis of cluster-specific genes: (*left*) line chart of expression changes; (*left-middle*) heatmap of specific gene expression; (*right-middle*) top five GO-BP terms per cluster; (*right*) bar graph of top five GO-BP terms ranked by -log_10_(*P-*value). *E,* Feature importance analysis quantifying variance in cell cluster proportions explained by experimental condition (PBS vs. OPCR-Lip), biological replicates, and residual factors. *F,* UMAP visualization of T-cell subclusters.


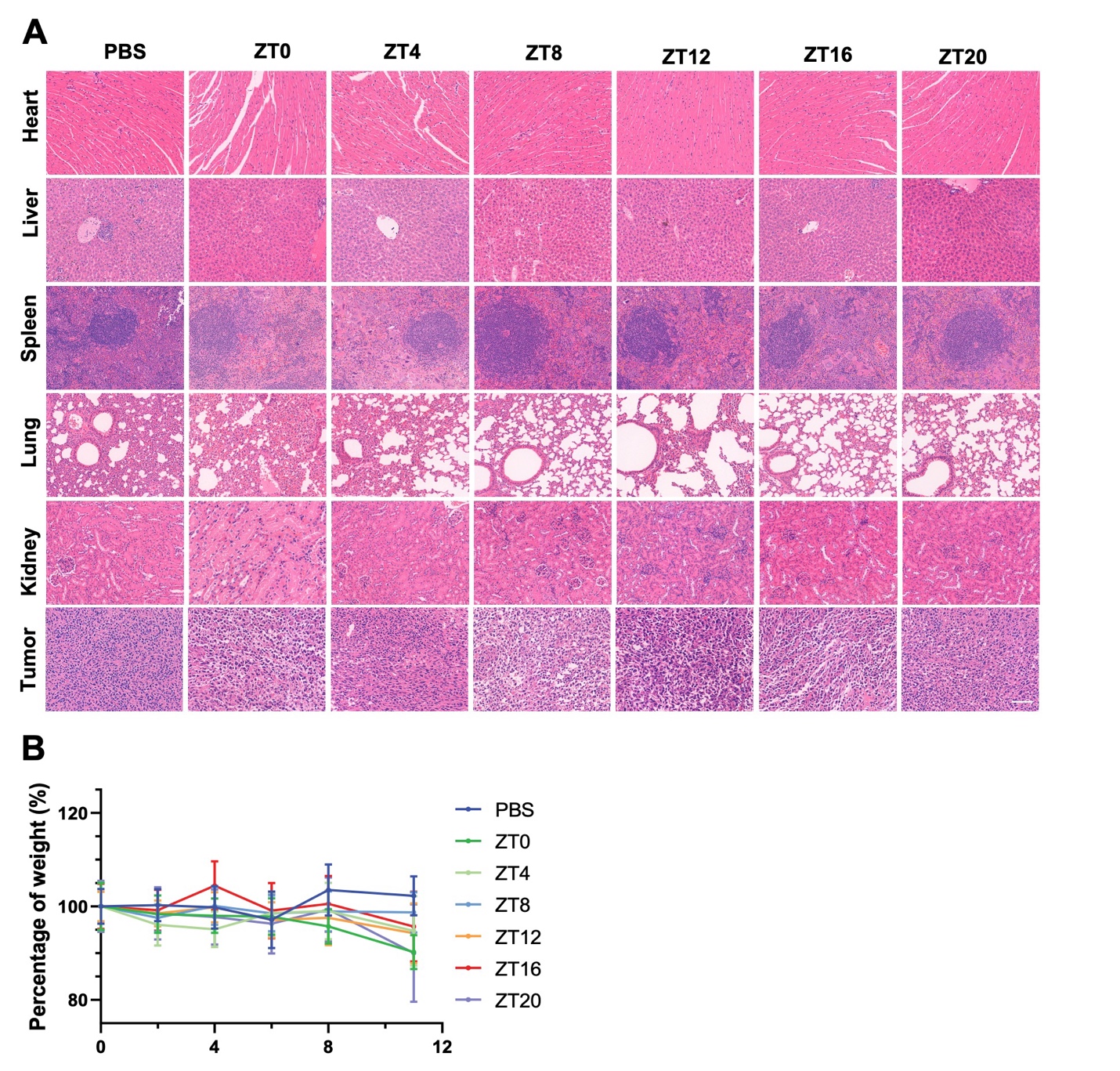


**Figure S13. Antitumor efficacy and systemic safety of circadian rhythm–based OPCR-Lip administration.** *A,* H&E staining of tissue sections from treated mice reveals histopathological changes. Scale bar: 50 μm. *B,* Body weight monitoring of treated mice (*n* = 3), indicating systemic tolerability.


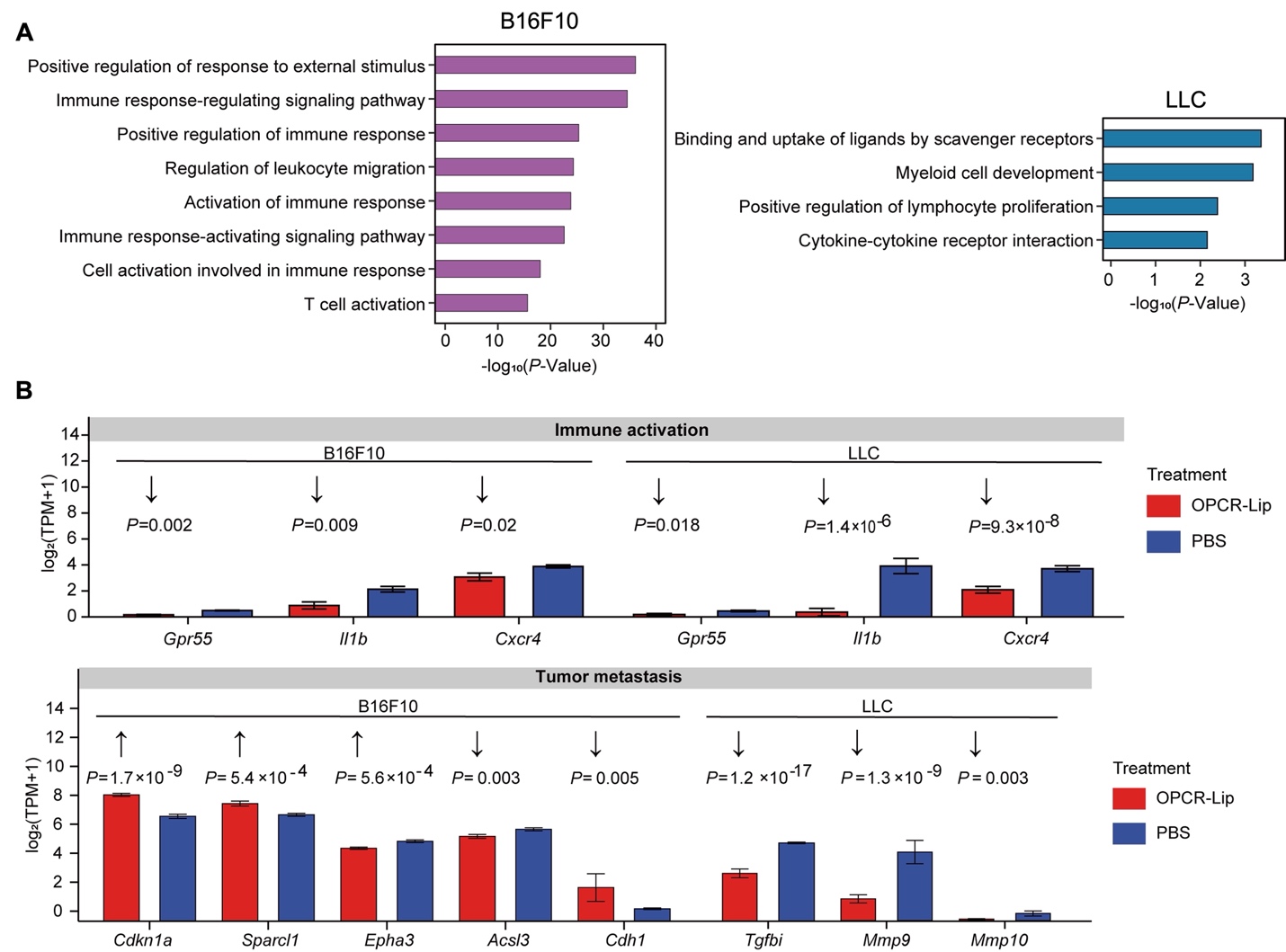


**Figure S14.** **Functional enrichment and immune gene** **expression analysis in OPCR-Lip–treated B16F10 and LLC tumors.** *A,* Representative functional enrichment terms derived from differentially expressed genes in B16F10 and LLC tumors following OPCR-Lip treatment. *B,* Expression changes in metastasis-associated genes and key immune activation marker genes. Arrows indicate expected regulatory trends and functional enhancements driven by OPCR-Lip. Data are presented as mean ± SD.

**Table S1. Mean survival times of 4T1 tumor-bearing mice treated with different formulations.**

| Groups | PBS | O-Lip | PCR-Lip | OCR-Lip | OPCD-Lip | OPCR-Lip |
| --- | --- | --- | --- | --- | --- | --- |
| Mean survival times  (days) | 17 | 22 | 19 | 25 | 34 | >36 |

**Table S2. List of 21 signature genes associated with OPCR-Lip-induced responses and their functional descriptions.**

| Gene symbol | Gene name | Functional description |
| --- | --- | --- |
| ***Perp*** | p53 apoptosis effector related to PMP22 | A direct downstream effector of p53 (69), *Perp* is activated in response to DNA damage. It promotes apoptosis by modulating mitochondrial apoptotic regulators such as the Bax/Bcl-2ratio (70). |
| ***Trp53tg5*** | transformation related protein 53 target 5 | A p53-responsive gene involved in DNA damage responses. *Trp53tg5* contributes to cell cycle arrest, apoptosis, and senescence following *TP53* activation (71,72)*.* |
| ***Chd5*** | chromodomain helicase DNA binding protein 5 | A chromatin remodeling factor that preserves genome stability through DNA repair regulation (73). *Chd5* overexpression inhibits tumorigenesis via p14/p53 and p16/RB signaling activation (74). |
| ***Gadd45b*** | growth arrest and DNA damage inducible beta | Rapidly upregulated upon genotoxic or stress stimuli, *Gadd45b* modulates DNA repair, cell cycle arrest, and apoptosis(75,76). It also participates in mitochondrial metabolism (77). |
| ***Hspb1*** | heat shock protein family B (small) member 1 | Regulates DNA repair pathways (78). Its expression is induced by genotoxic stress as part of the heat shock protein response (79). |
| ***Hic1*** | hypermethylated in cancer 1 | A critical regulator of DNA repair and genomic stability, *HIC1* integrates epigenetic control with damage responses. Its loss contributes to tumorigenesis via impaired repair (80,81). |
| ***Bnipl*** | BCL2 interacting protein like | Modulates immunogenic cell death (ICD) through mitochondrial homeostasis. *Bnipl*-mediated mitochondrial damage triggers release of DAMPs such as ATP and HMGB1(82,83). |
| ***Nfkb1*** | nuclear factor kappa B subunit 1 | Induces apoptosis via death receptor upregulation (e.g., Fas) (84), indirectly modulating ICD pathways (85). |
| ***Rela*** | RELA proto-oncogene, NF-kB subunit | A central NF-κB transcription factor, *RelA* regulates inflammatory cytokines (e.g., TNF-α, IL-2), influencing the immunogenic microenvironment (86). |
| ***Ccl20*** | C-C motif chemokine ligand 20 | A key ICD-related gene (87), *CCL20* promotes anti-tumor immunity by enhancing tumor cell immunogenicity (88). |
| ***Dnajc12*** | DnaJ heat shock protein family (Hsp40) member C12 | Downregulation of *Dnajc12* may increase chemotherapy resistance by inhibiting apoptosis and ferroptosis, thereby impairing ICD induction (89). |
| ***Tnfrsf1b*** | TNF receptor superfamily member 1B | Downregulation shifts TNF signaling towards apoptosis and inflammation, enhancing DAMP release and ICD (90). |
| ***Map3k1*** | mitogen-activated protein kinase kinase kinase 1 | Regulates inflammation and stress signaling, potentially modulating DAMP release during ICD (91). |
| ***Ccl21a*** | C-C motif chemokine ligand 21 | Facilitates immune cell migration to lymph nodes, supporting anti-tumor responses (92). |
| ***Cpm*** | carboxypeptidase M | Upregulated in macrophages during T cell activation, suggesting a role in immune activation (93). |
| ***Ccl20*** | C-C motif chemokine ligand 20 | Enhances therapeutic sensitivity by promoting ILC3-mediated immune responses (94). |
| ***Ccl22*** | C-C motif chemokine ligand 22 | Downregulation reduces Treg recruitment and impairs immune escape mechanisms (e.g., PI3K/NF-κB signaling), thereby enhancing anti-tumor immunity (95). |
| ***Ccr8*** | C-C motif chemokine receptor 8 | Inhibition or downregulation improves the immune microenvironment by targeting tumor-infiltrating Tregs (96). |
| ***Cd40lg*** | CD40 ligand | Sustained immunotherapy may lead to selective downregulation by tumor cells in response to immune pressure (97). |
| ***Ctla2b*** | cytotoxic T lymphocyte-associated protein 2 beta | A cysteine protease inhibitor expressed by activated T cells and mast cells, possibly modulating immune homeostasis (98). |
|  |  |  |
